# Bifunctional trehalase FsTreA coordinates intracellular mobilization and extracellular utilization of trehalose to modulate virulence in *Fusarium sacchari*

**DOI:** 10.1128/aem.00697-26

**Published:** 2026-05-14

**Authors:** Yuejia Chen, Yueying Zhao, Gengzhong Cheng, Yuming Lin, Qianmin Liang, Lianke Zhu, Liuting Huang, Xinyi Lin, Ziting Yao, Chengwu Zou

**Affiliations:** 1 State Key Laboratory for Conservation and Utilization of Subtropical Agro-Bioresources, Ministry and Province Sponsored Center of Collaborative Innovation for Sugarcane Industry, College of Agriculture, Guangxi University 12664, Nanning, China; 2 Plant Protection Research Institute, Guangxi Academy of Agricultural Science, Nanning, China; The University of Arizona, Tucson, Arizona, USA

**Keywords:** sugarcane, Pokkah Boeng Disease, *Fusarium sacchari*, bifunctional trehalase, virulence

## Abstract

*Fusarium sacchari* causes devastating Sugarcane Pokkah Boeng Disease by infecting tissues through conidia that enter via micro-wounds or stomata on the leaf surface, making conidial germination a critical step in its pathogenicity. Here, we identified a trehalase, FsTreA, which contains not only the essential trehalase structural domain characteristic of neutral trehalases but also features an N-terminal signal peptide. *FsTreA* exhibits the highest transcriptional level among all trehalases during conidial germination. Disruption of *FsTreA* significantly elevated intracellular trehalose accumulation in conidia, confirming its role in intracellular mobilization. However, ΔFsTreA showed accelerated trehalose mobilization rates during germination, coinciding with enhanced conidial germination, and increased conidial yield. This was attributed to functional compensation by two other trehalases, FsNth1 and FsAth1. Furthermore, we found that FsTreA predominantly localizes to the cell surface and possesses the ability to mobilize extracellular trehalose. Moreover, the virulence of *FsTreA* mutant strain was positively correlated with the capability for extracellular trehalose mobilization. In conclusion, our results demonstrate that the bifunctional trehalase FsTreA coordinates intracellular and extracellular trehalose utilization to modulate virulence in *F. sacchari*.

**IMPORTANCE:**

Sugarcane Pokkah Boeng Disease, caused by *Fusarium sacchari*, poses a severe threat to global sugarcane production. This study represents the first identification of the bifunctional trehalase FsTreA in *F. sacchari*, which breaks the traditional dichotomous classification framework of traditional fungal trehalases. FsTreA harbors both the GH37 catalytic domain characteristic of neutral trehalases and an N-terminal signal peptide conferring the secretory trait of acid trehalases. This enzyme participates in intracellular trehalose mobilization and is simultaneously secreted extracellularly to hijack host-derived trehalose as a carbon source, constituting a distinctive infection strategy that provides new insights into host-pathogen interaction mechanisms. These findings advance the theoretical understanding of fungal metabolic regulation, holding significant scientific and practical value for safeguarding the security of the global sugarcane industry.

## INTRODUCTION

Sugarcane (*Saccharum spp*.) is a fundamental raw material for sugar production (1). However, the sugarcane industry has long been threatened by various diseases. Pokkah Boeng Disease (PBD), a prominent epidemic fungal disease, significantly reduces both the yield and sucrose content of sugarcane (2–4). To date, studies on PBD have predominantly focused on pathogen identification, germplasm resistance, and the impacts of environmental factors on disease progression (5–7). Nevertheless, identifying the causal agent and delineating its pathogenesis remain strategic imperatives for effective PBD control.

*F. sacchari* is the primary pathogen responsible for PBD in China, invading the host through micro-wounds via conidia to establish infection. Consequently, conidial germination constitutes the cardinal determinant of successful host colonization. Trehalose is a stable non-reducing disaccharide composed of two glucose units linked in an α,α−1,1 configuration (8). It is widely present in microorganisms such as bacteria and fungi (9, 10). In fungi, trehalose serves not only as a key energy-storage compound (accounting for up to 15% in spores) (11, 12), but also provides a carbon source for germination (13). Additionally, it helps spores resist environmental stress prior to germination. In yeast, trehalose in spores is essential for survival during long-term desiccation (14). In arbuscular mycorrhizal fungi, trehalose aids recovery in response to heat shock and arsenate stress (15). Fungi urgently need to mobilize trehalose during the critical process of conidial germination (16).

Trehalose mobilization depends on trehalases (17). These enzymes are crucial for fungal carbon source metabolism, stress adaptation, and pathogenic processes (18, 19). Currently, research on genes encoding trehalases has established a substantial foundation. They also play important roles in metabolic regulation. In *Saccharomyces cerevisiae*, dual deletion of *NTH1* and *ATH1* elevates intracellular trehalose concentrations while impairing conidial germination and carbon utilization (20). The rice blast pathogen *Magnaporthe oryzae* requires *NTH1* for host colonization; deletion mutants exhibit attenuated pathogenicity (21). In *Candida albicans*, the gene encoding the cell wall-associated acid trehalase, *Atc1*, has emerged as a promising therapeutic target for novel antifungal agents. The gene-knockout mutant exhibited impaired hyphal and pseudohyphal formation capabilities, which consequently led to a significant reduction in its pathogenicity (22, 23). However, the regulatory mechanism of trehalose metabolism, especially the role of trehalase, remains unclear in *F. sacchari*.

In the present study, we identified a bifunctional trehalase, FsTreA, which coordinates trehalose mobilization in conjunction with neutral trehalase FsNth1 and acid trehalase FsAth1 through reciprocal compensation, thereby regulating germination and sporulation. Additionally, FsTreA utilizes extracellular trehalose from sugarcane as an energy source, thereby enhancing fungal virulence. These findings demonstrate that trehalase is essential for the development and virulence of *F. sacchari* through trehalose mobilization.

## MATERIALS AND METHODS

### Strains and culture conditions

The wild-type strain CNO-1 of *F. sacchari* was used for the construction of *FsTreA* mutant strains (24). All strains were grown at 28°C on potato dextrose agar (PDA) plates for 7 days to assess their phenotypic traits. Sporulation was measured as described previously (25). For conidial germination assays, spores were harvested from 7-day-old colonies on PDA plates and then inoculated in PDB liquid medium at 28°C with shaking at 150 rpm for 6 h. The *Escherichia coli* strains DH5α and BL21(DE3) were used for plasmid construction and expressing trehalases, respectively. They were cultured on lysogeny broth medium at 37°C.

### Bioinformatic analysis

Signal peptides (SP) were identified by the SignalP 5.0 server (https://services.healthtech.dtu.dk/services/SignalP-5.0/). Protein domains were predicted by Pfam (http://pfam.xfam.org/). The homologous sequences were compared using BLASTp in the NCBI database, and sequences with high similarity were selected. A phylogenetic tree was constructed using MEGA 7 with the maximum likelihood method, and iTOL v7 for visualization (https://itol.embl.de/) (26).

### RNA extraction and quantitative real-time reverse transcription PCR

The total RNA from each strain was extracted using the RNA extraction kit (TaKaRa, Beijing, China) according to the manufacturer’s protocol. First-strand cDNA synthesis was performed using the FastQuant RT Kit (TaKaRa, Beijing, China). Quantitative real-time PCR was conducted using the SuperReal PreMix Plus (TaKaRa, Beijing, China) with the target gene primer pairs (Table S1) and 18S rRNA as an internal control (27). The relative expression levels were calculated using the 2^−∆∆Ct^ method (28). The data are presented as the mean ± SE from three independent biological replicates.

### Trehalase-encoding gene-related mutant strain construction

To knock out *FsTreA*, *FsNth1*, and *FsAth1*, deletion fragments were amplified using fusion PCR with the primer pairs FsTreA-A/B, FsNth1-A/B, and FsAth1-A/B, respectively (Table S1). The hygromycin resistance gene (*Hph*) was employed as a selection marker (29). The amplified fragments were transformed into protoplasts of the wild-type strain CNO-1 using a polyethylene glycol (PEG, MW 3350, 40% [wt/vol])-mediated method (30), resulting in the trehalase-encoding gene deletion mutants, named ΔFsTreA, ΔFsNth1, and ΔFsAth1, respectively. For the complementation strains, full-length *FsTreA* fragments, including promoter sequences, were amplified using the primer pair C-FsTreA-F/C-FsTreA-R (Table S1). These fragments were cloned into the pCPXG418 vector using the pEASY-Basic Seamless Cloning and Assembly Kit (Transgen Biotech, Beijing, China) and then transformed into the protoplasts of Δ*FsTreA*. The overexpression strains were developed by introducing additional copies of *FsTreA* into the CNO-1 protoplasts, which were then selected on media supplemented with the appropriate antibiotics.

### Trehalose content and trehalase activity quantification

For the detection of trehalase activity in fungal strains, 1 mL of the spore suspension (1 × 10^7^ conidia/mL) of each strain was inoculated into 100 mL PDB liquid medium and cultured for 3 days (31). Mycelia were filtered through four layers of gauze, and the remaining culture medium was centrifuged to collect spores for testing. For the detection of trehalose content in the host tissues, 1 cm of tissue around the inoculation site was collected, ground in liquid nitrogen, and then tested. The trehalase activity and trehalose content were measured using the Solarbio (Beijing, China) Trehalase (THL) Activity Assay Kit and Trehalose Content Assay Kit, respectively, following the instructions provided with each kit.

### Yeast signal sequence trap experiment

Functional validation of the predicted signal protein was conducted with a yeast secretion system (32). DNA fragments encoding SP of FsTreA were amplified using primers pSUC2-SP^FsTreA^-F/R and introduced into pSUC2 at the N-terminal of the invertase. The pSUC2-SP^FsTreA^ vector was transformed into the yeast strain YTK12 and screened on CMD-W medium. Positive colonies were replica plated on YPRAA medium plates to detect invertase secretion and subjected to the 2,3,5-triphenyltetrazolium chloride (TTC) color reaction assay. YTK12 was transformed with pSUC2-SP^Avr1b^, and the empty pSUC2 vector was used as a positive and negative control, respectively.

### Subcellular localization analysis

To construct the plasmids of FsTreA-mCherry, the full-length of *FsTreA* was amplified and inserted into pCPXG418-mCherry, which was digested by *Not* I. Then, it was transformed into CNO-1, resulting in an FsTreA fluorescence-labeled strain, named FsTreA::mCherry (33). To confirm the expression of the mCherry fusion proteins, hyphae were cultured on PDA for 48 h, and mCherry signals were observed and photographed using an Olympus DP70 microscope (Olympus, Tokyo, Japan). To further verify the location of FsTreA, the cell wall and cytoplasmic proteins of FsTreA::mCherry were extracted using protein Extract Kit (Beyotime Biotech, ShangHai, China). Western blotting was performed on the cell wall and cytoplasmic fractions of each strain using Anti-mCherry (Sanying Biotech, WuHan, China).

### Virulence assay

To assess the virulence of FsTreA mutants, sugarcane plants at the five-leaf stage were inoculated with a 300 µL volume of conidial suspensions (1 × 10^4^ conidial/mL) from each strain. Disease severity was evaluated 14 days post-inoculation (dpi), and the disease severity index (DSI) was calculated using a symptom severity scale (25). The DSI was determined using the formula DSI = 100 × (Σ score/5*N*), where *N* represents the number of observed seedlings (*N* = 50). Each assay was replicated three times. Sugarcane leaves were scraped with a blade and then inoculated with mycelial plugs (6 mm in diameter). The leaves were then incubated at 28°C (34). The amount of leaf necrosis was observed and photographed after 2 days. Disease assays on transgenic sugarcane plants were conducted using the methodology described previously.

### Expression and purification of trehalases

FsNth1, FsTreA, and FsAth1 (without SP) were amplified and cloned into pGEX-GT, digested with *EcoR* I and *BamH* I, respectively. Three trehalase recombinant proteins were expressed in *E. coli* strain BL21(DE3). Expression was induced by adding 0.2 mM IPTG for 12 h at 16°C. For protein extraction, the supernatant was collected by centrifugation at 5,000 rpm for 30 min. Trehalases were purified using glutathione S-transferase (GST) resin (Beyotime Biotech, Shanghai, China) following the manufacturer’s instructions. The purification protein samples were concentrated using centrifugal filter devices with 50 KDa (Sangon Biotech, Shanghai, China). Recombinant protein expression was verified by concentration determination using the bicinchoninic acid (BCA) method and by western blot analysis using the anti-GST antibody (Abcam, Shanghai, China).

### Statistical analysis

GraphPad Prism 9.0 was used for the statistical analysis of the numerical data. Significant differences were analyzed using two-tailed Student’s *t*-test or one-way analysis of variance followed by Duncan’s multiple comparisons test. Asterisks are used to indicate *P* < 0.05 (*), *P* < 0.01 (**), and *P* < 0.001 (***). Different letters indicate significant differences at *P* < 0.05.

## RESULTS

### Identification of trehalases in *F. sacchari*

**Fig 1:**
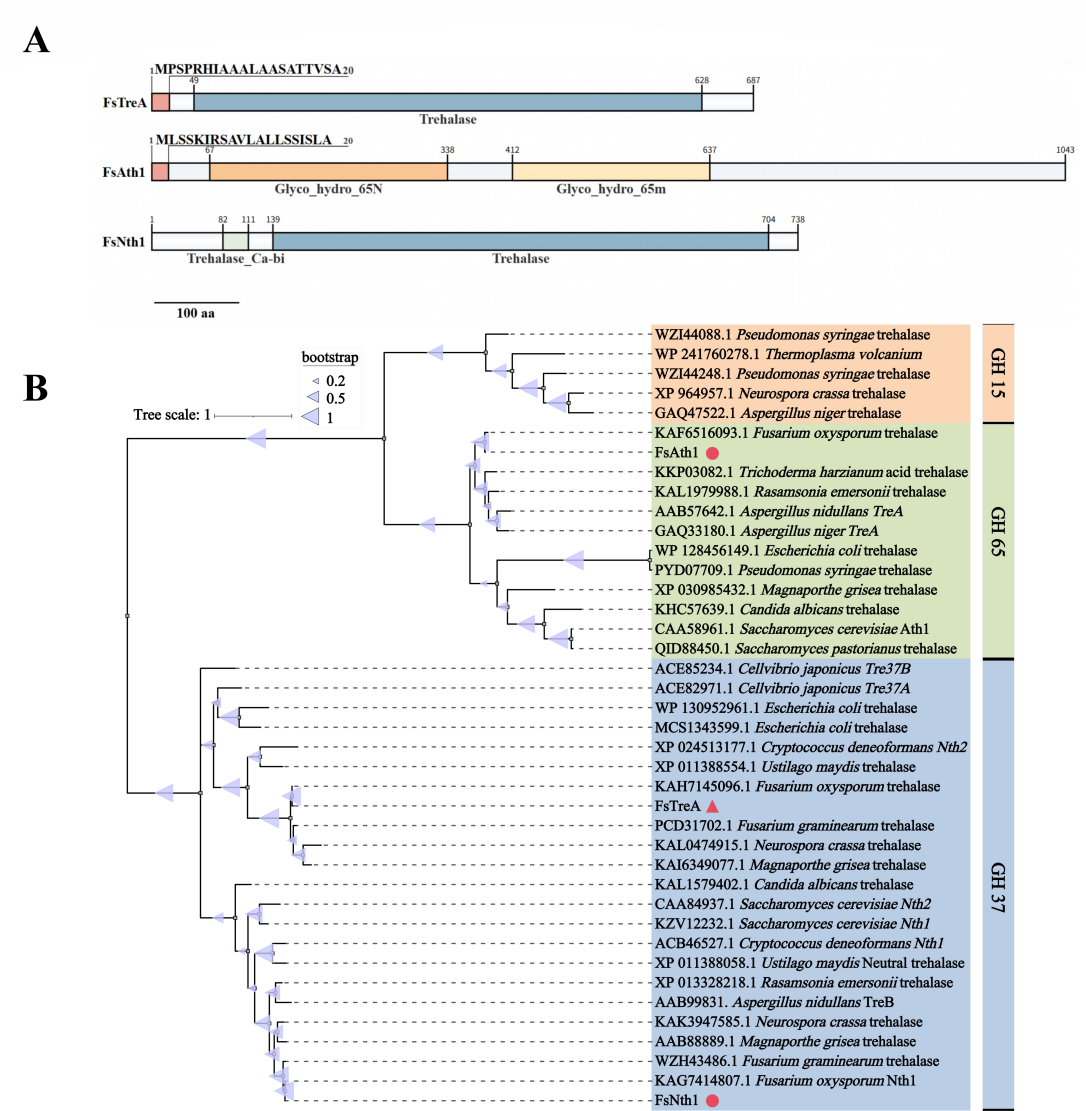
FsTreA, FsAth1, and FsNth1 are trehalases. (**A**) Prediction of the signal peptide and domain architecture for the three trehalases in *F. sacchari*. (**B**) Phylogenetic tree of the three trehalases constructed using the maximum likelihood method with MEGA 7. Colored stripes represent the protein family to which the corresponding sequences belong.

Trehalose mobilization serves as a critical energy source to power conidial germination. To identify trehalases in *F. sacchari*, amino acid sequences of Nth1 and Ath1 from *S. cerevisiae* were used as queries in BLAST searches against the genome of *F. sacchari* strain CNO-1. FsAth1 (PX557839) and FsNth1 (PX557838) were identified, with similarities of 49.7% and 47.9%, respectively. Notably, we discovered a novel trehalase, FsTreA (PX557837), which contains not only the essential trehalase domain characteristic of neutral trehalases but also possesses an N-terminal signal peptide specific to acid trehalases (Fig. 1A). Signal peptide prediction using SignalP 5.0 revealed that FsTreA possesses a 20-amino acid signal peptide with a predicted cleavage site between Ala20 and Leu21. Compared to other trehalases, FsTreA exhibits the lowest sequence similarity to *S. cerevisiae* Nth1 and Ath1, with merely 30.3% and 20.9% identity, respectively. Phylogenetic analysis confirmed that FsTreA constitutes a distinct clade, separate from both neutral and acid trehalases. Despite the low similarity, FsTreA and FsNth1 are both members of the GH37 glycoside hydrolase family. FsTreA is phylogenetically more closely related to neutral trehalases than to acidic trehalases (Fig. 1B), suggesting functional conservation within the neutral enzyme lineage.

### FsTreA exhibits the highest expression compared to other trehalases during conidium germination

**Fig 2:**
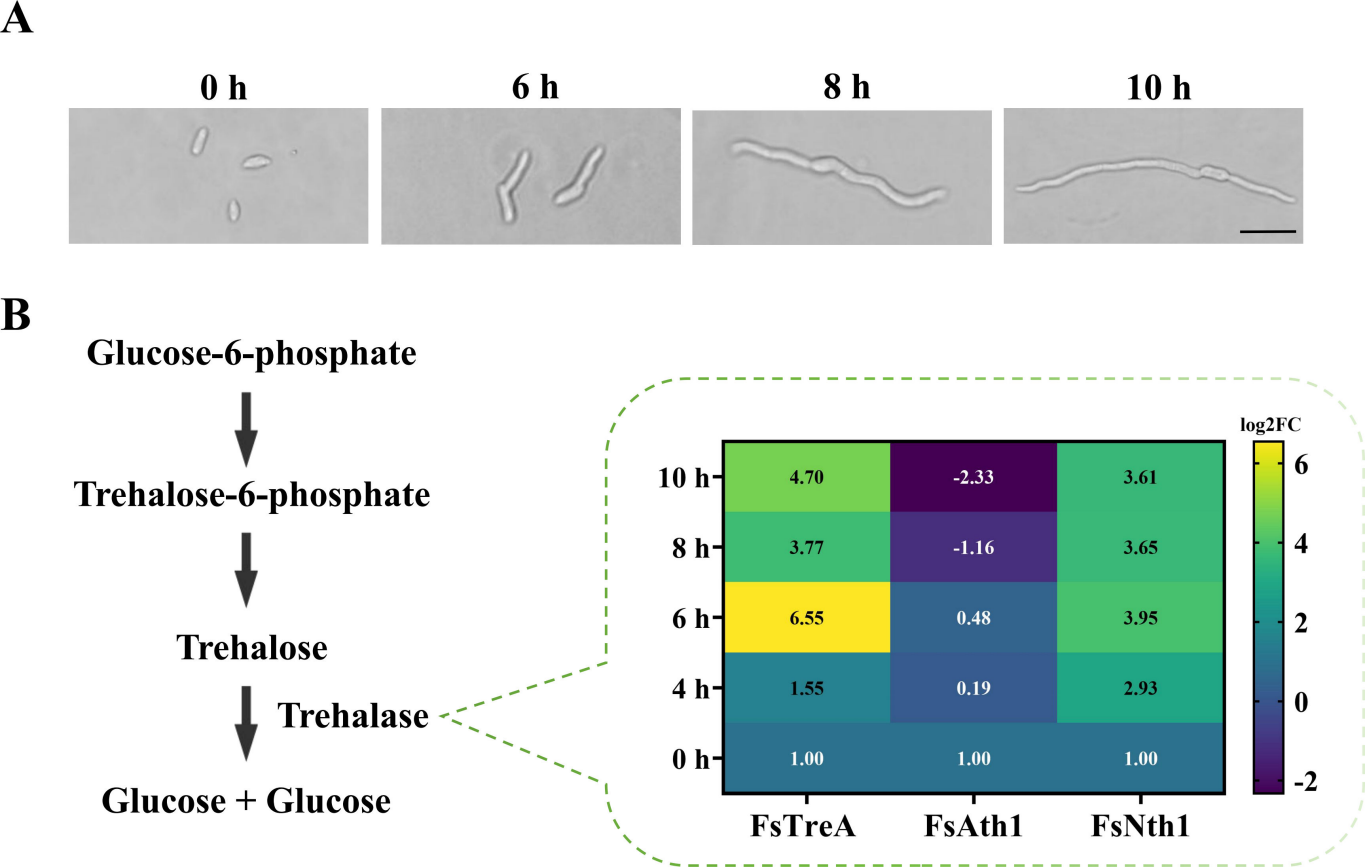
*FsTreA* exhibits the highest expression during conidial germination. (**A**) Development of conidial germination and hyphae. The spores of CNO-1 were inoculated in PDB medium, and representative pictures were captured at 0 h, 6 h, 8 h, and 10 h. Scale bar = 25 µm. (**B**) Relative expression of *FsTreA*, *FsAth1*, and *FsNth1* during conidial germination and hyphal morphogenesis. The transcript level of conidia was set to a value of 1.0, yellow indicates high expression, and purple indicates low expression.

To investigate the roles of these three trehalases in *F. sacchari*, we examined the transcriptional levels of trehalases across different stages of conidial germination, obtaining expression profiles of *FsTreA*, *FsAth1*, and *FsNth1* during conidial germination (6 h) and hyphal morphogenesis (8 and 10 h) (Fig. 2A). Transcriptomic profiling revealed that *FsTreA* and *FsNth1* were significantly upregulated compared to *FsAth1* during this process. *FsTreA* exhibited peak expression at 6 h, at levels 1.5-fold higher than *FsNth1*, whereas its expression stabilized during hyphal formation. In contrast, *FsAth1* expression remained unchanged throughout (Fig. 2B). These findings suggest that when genes encoding all three trehalases coexist, *FsTreA* and *FsNth1* coordinately regulate the development of *F. sacchari*, with *FsTreA* serving as the primary regulator during conidial germination.

### FsTreA regulates *F. sacchari* sporulation and germination

**Fig 3:**
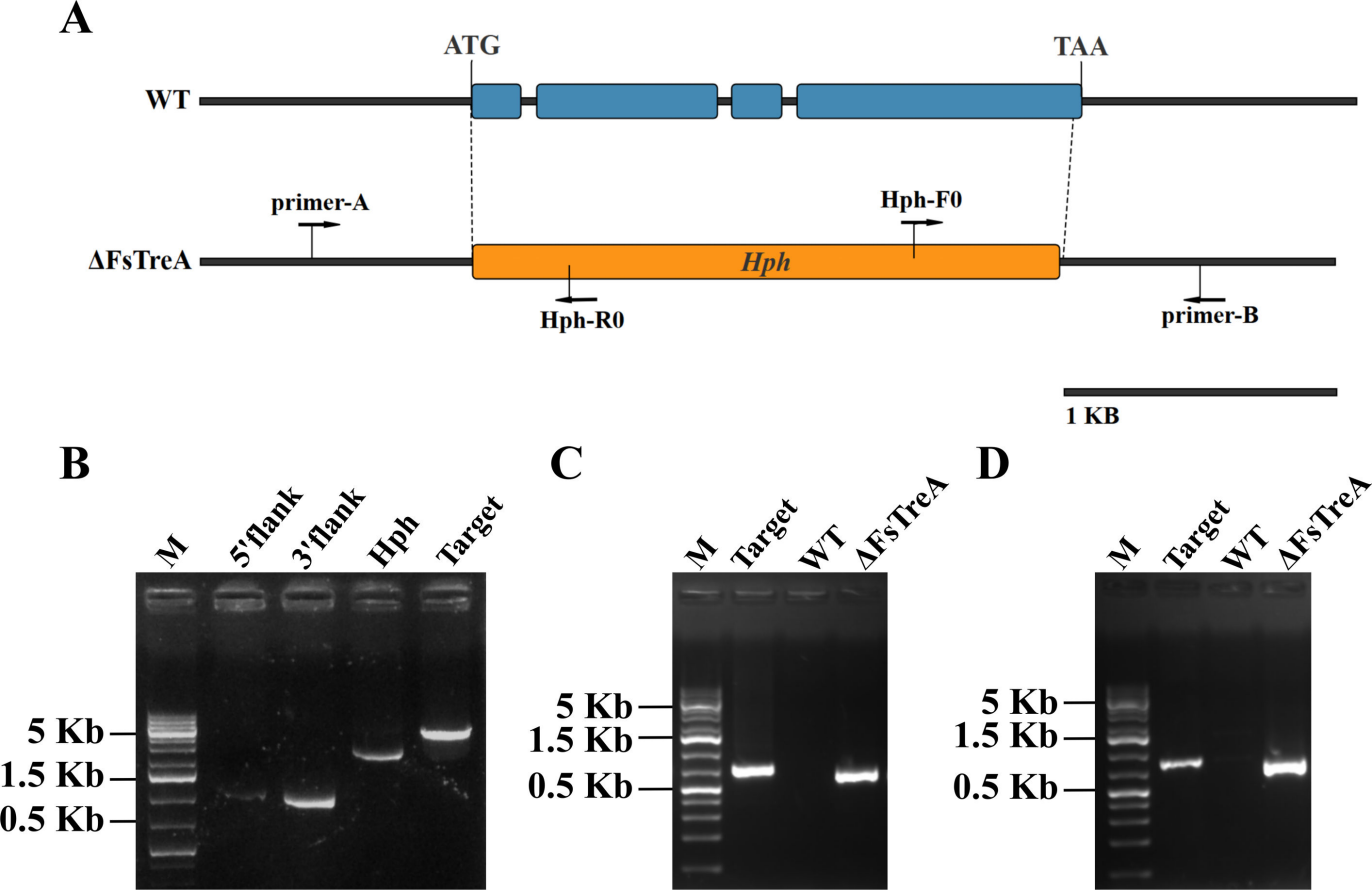
Generation of *FsTreA* deletion mutant strains. (**A and B**) *FsTreA* gene locus and gene replacement construct. (**C and D**) *FsTreA* deletion mutants were validated by PCR with primer A/Hph-R0 and Hph-F0/primer B, respectively. Phenotypic traits indicated that the deletion of *FsTreA* did not affect the growth of *F. sacchari* but significantly increased conidial yield and germination rate by 36% and 44%, respectively, compared to the wild type. Conversely, the overexpression of *FsTreA* enhanced the development of *F. sacchari*, increasing colony diameter by 8.7% compared to the wild type. Complementation of *FsTreA* restored phenotypes to wild-type levels without any statistically significant difference (Fig. 4). These results suggest that *FsTreA* modulates sporulation and germination in *F. sacchari*.

To investigate the biological role of *FsTreA* in *F. sacchari*, we generated the *FsTreA* deletion mutant Δ*FsTreA* by replacement with the hygromycin resistance gene (Fig. 3; Fig. S3). The reintroduction of the wild-type copy of *FsTreA* into Δ*FsTreA* constructed the complementation strain, designated C-Δ*FsTreA*, and the *FsTreA* overexpression mutant strain, designated O-*FsTreA* (Fig. S1 and S2). The wild-type strain CNO-1, Δ*FsTreA*, O-FsTreA, and C-Δ*FsTreA* were used for phenotypic analyses.

**Fig 4:**
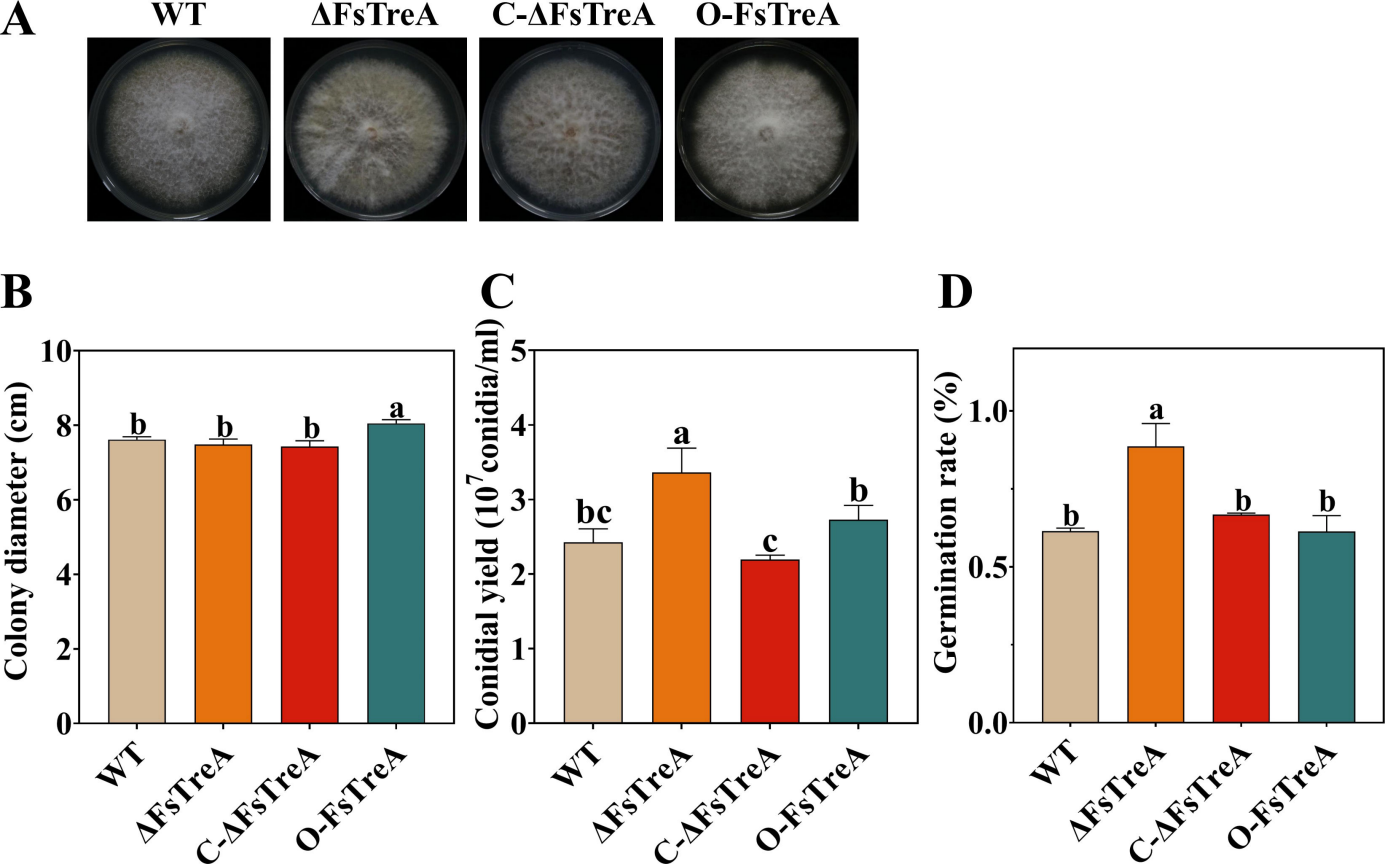
*FsTreA* regulates sporulation and germination in *F. sacchari*. (**A**) Phenotypes of *FsTreA*-related mutants. All strains were inoculated on PDA plates at 28°C for 7 days. (**B**) Colony diameters of all strains were measured. (**C**) Statistics of conidial yield for all strains. Conidia were harvested from 7-day-old colonies on PDA plates. (**D**) Conidial germination rates in PDB at 28°C for 6 h. All experiments were replicated three times. Values represent means ± SE of three biological replicates. Different letters indicate significant differences at *P* < 0.05 as measured by Duncan’s multiple comparisons test.

### The mobilization of intracellular trehalose is coordinately regulated by FsTreA, FsNth1, and FsAth1 through a reciprocal compensatory mechanism

**Fig 5:**
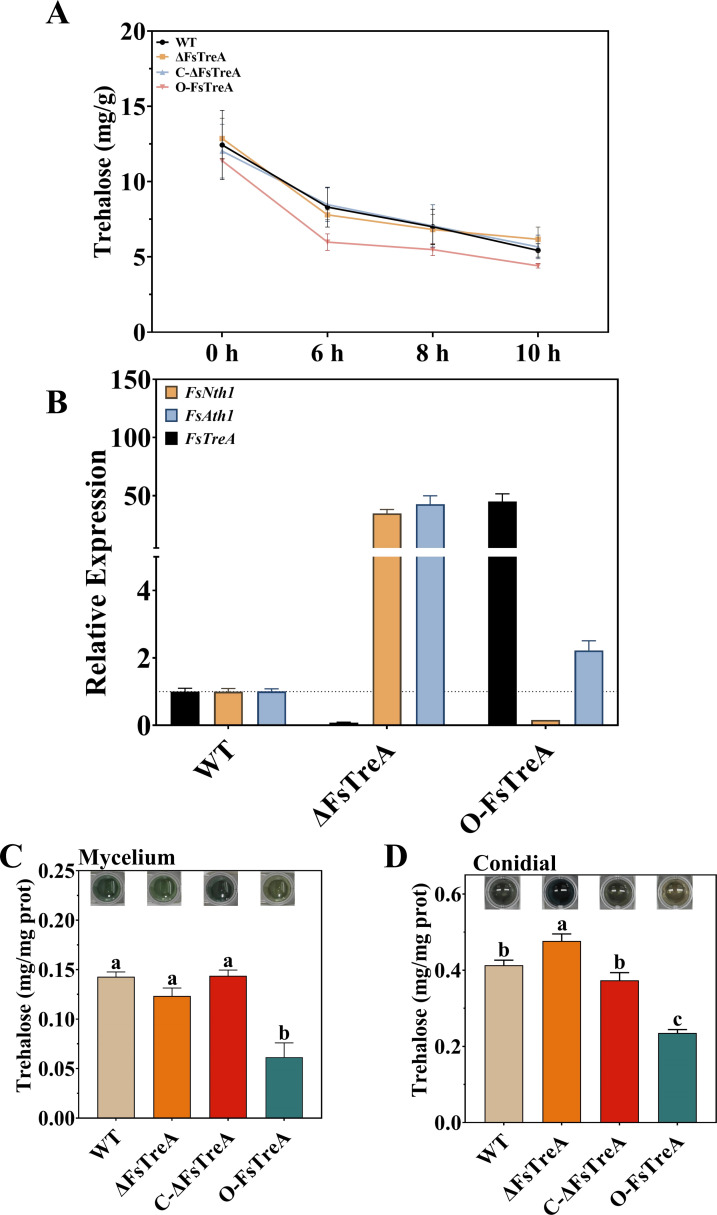
*FsTreA*, *FsNth1*, and *FsAth1* cooperatively regulate intracellular trehalose mobilization in *F. sacchari*. (**A**) Trehalose quantification content in *FsTreA* mutants across developmental stages of germination and hyphae (on a fresh weight basis). (**B**) Expression patterns of three genes encoding trehalases in *FsTreA* mutants. The expression of three genes was measured by RT-qPCR (2^−ΔΔct^ method) with 18S rRNA as an internal reference. Transcript levels of the three genes in the wild-type were normalized to 1.0. (**C**) Trehalose quantification in the mycelium of *FsTreA* mutants. (**D**) Trehalose quantification in conidia of *FsTreA* mutants. Values represent means ± SE of three biological replicates. Different letters indicate significant differences at *P* < 0.05 as measured by Duncan’s multiple comparisons test.

Fungi require trehalases to mobilize intracellular trehalose for conidial germination. However, our results demonstrate that the deletion of *FsTreA* enhances both germination and conidiation, as shown in Fig. 4. To investigate this phenomenon, we measured the trehalose mobilization capacity and intracellular content of trehalose in *FsTreA*-related mutants during conidial germination and hyphal morphogenesis. These data reveal that overexpression of *FsTreA* significantly accelerates intracellular trehalose consumption in *F. sacchari*, while the trehalose accumulation in Δ*FsTreA* conidia is substantially higher than that in WT conidia, thus conclusively establishing that FsTreA plays an essential role in trehalose mobilization (Fig. 5A and C). However, Δ*FsTreA* exhibited faster trehalose mobilization rates than WT (Fig. 5A and D), with the trehalose accumulation levels in the hyphae showing a non-significant difference. Since *F. sacchari* harbors three trehalase isozymes, this accelerated mobilization phenotype likely points to functional compensation by the remaining trehalases (FsAth1 and FsNth1) following *FsTreA* deletion. To verify this hypothesis, we quantified the expression levels of trehalase genes Δ*FsTreA* and O-FsTreA. The disruption of *FsTreA* significantly increased the expression of *FsAth1* and *FsNth1* to 42- and 34-fold of the wild-type levels, respectively (Fig. 5B). In contrast, the overexpression of *FsTreA* sustained the induction of *FsAth1* but nearly eliminated FsNth1 expression. These findings indicate that FsTreA, FsNth1, and FsAth1 cooperatively regulate intracellular trehalose mobilization in *F. sacchari* through a reciprocal compensatory manner.

### FsTreA exhibits secretory activity similar to acid trehalases

**Fig 6:**
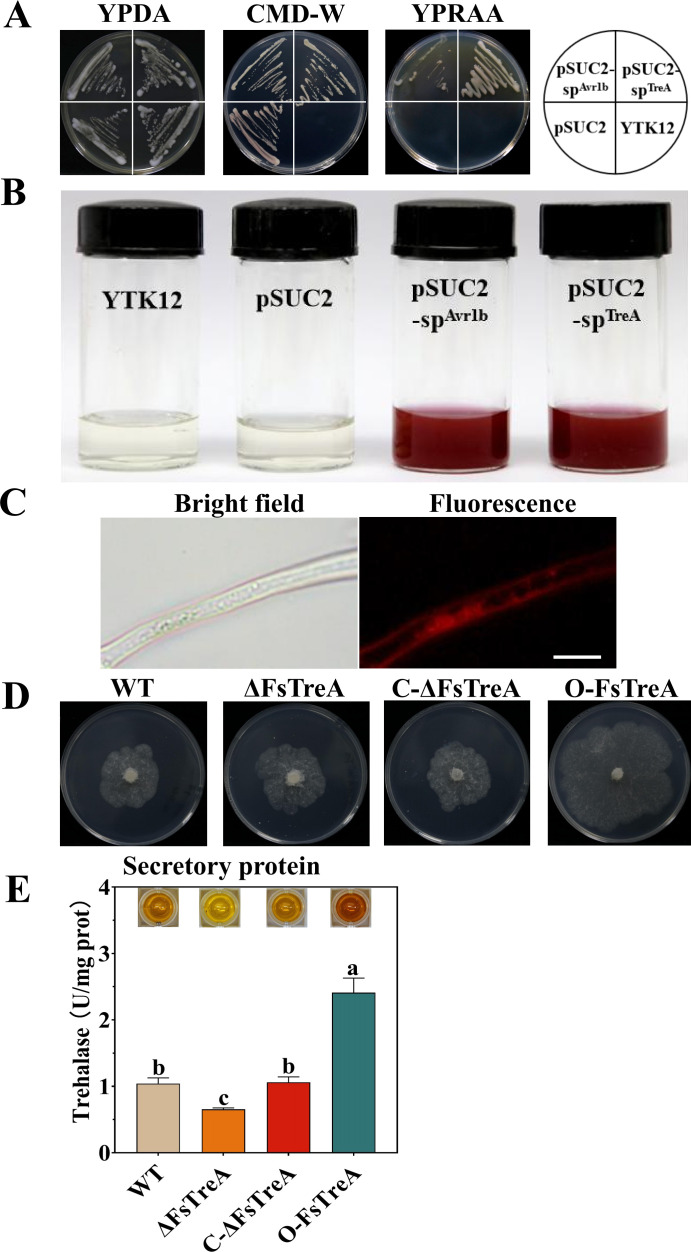
FsTreA demonstrates functional attributes of an acid trehalase in *F. sacchari*. (**A**) Validation of the N-terminal signal peptide of FsTreA by the yeast secretion system. The SP was fused to mature yeast invertase. The YTK12 strain is confined to yeast extract peptone dextrose adenine (YPDA) medium for growth. Those with the SP-fused vector thrive on YPDA, CMD-W, and YPRAA media. (**B**) Secreted invertase can reduce triphenyltetrazolium chloride (TTC) to red formazan. The pSUC2-sp^Avr1b^ was used as the positive control, and the empty pSUC2 vector as the negative control, both cultured on YPDA plates for 2 days. (**C**) Subcellular location of FsTreA in *F. sacchari*. FsTreA::mCherry was constructed and transformed into the wild-type strain CNO-1, which was cultured at 28°C for 2 days. Hyphal samples were examined by fluorescence microscopy. Scale bar = 10 µm. (**D**) Phenotypes of *FsTreA* mutants on minimal medium (MM) plates with trehalose as the sole carbon source for 3 days. (**E**) Trehalase activity quantification of secreted protein in *FsTreA* mutants. The conidial suspensions of all strains were harvested from PDA plates after 7 days and incubated in PDB medium for 3 days. The hyphae of all strains were harvested for extracting secreted protein. Values represent means ± SE of three biological replicates. Different letters indicate significant differences at *P* < 0.05 as measured by Duncan’s multiple comparisons test.

Similar to FsAth1, FsTreA possesses an N-terminal signal peptide characteristic of acid trehalases. To validate the secretory capacity of FsTreA, a yeast signal trap assay was performed. The coding sequence of the N-terminal region of FsTreA (MPSPRHIAALAASATTVSA) was cloned into the yeast invertase vector pSUC2, and then all the constructs were transformed into the yeast strain YTK12. The strain containing *PsAvr1b* was used as the positive control in this assay. Only constructs containing the fused FsTreA and PsAvr1b could grow on YPRAA medium and catalyze the conversion of TTC to the red product triphenylformazan (Fig. 6B). In contrast, YTK12 and the strain carrying the pSUC2 vector used as a negative control did not change the color of the culture. These results confirmed that FsTreA exhibits secretory activity.

Furthermore, we determined the subcellular localization of FsTreA. As expected, the red fluorescence signal of the FsTreA-mCherry strain was predominantly observed at the cell surface (Fig. 6C; Fig. S6). Subsequently, we inoculated FsTreA mutants on MM plates with trehalose as the sole carbon source. The colony diameter of O-FsTreA was significantly increased, but Δ*FsTreA* showed no difference compared to WT. However, when cultured for 7 days, the growth of the Δ*FsTreA* was significantly inhibited compared to WT (Fig. 6D; Fig. S7A). Additionally, we performed enzymatic assays on secreted protein, which revealed significantly reduced trehalase activity in Δ*FsTreA* mutants, while O-*FsTreA* exhibited markedly higher activity than WT (Fig. 6E). This demonstrates that FsTreA functions as a secreted acid trehalase capable of mobilizing extracellular trehalose.

### FstreA positively regulates virulence by mobilizing trehalose within sugarcane tissues

**Fig 7:**
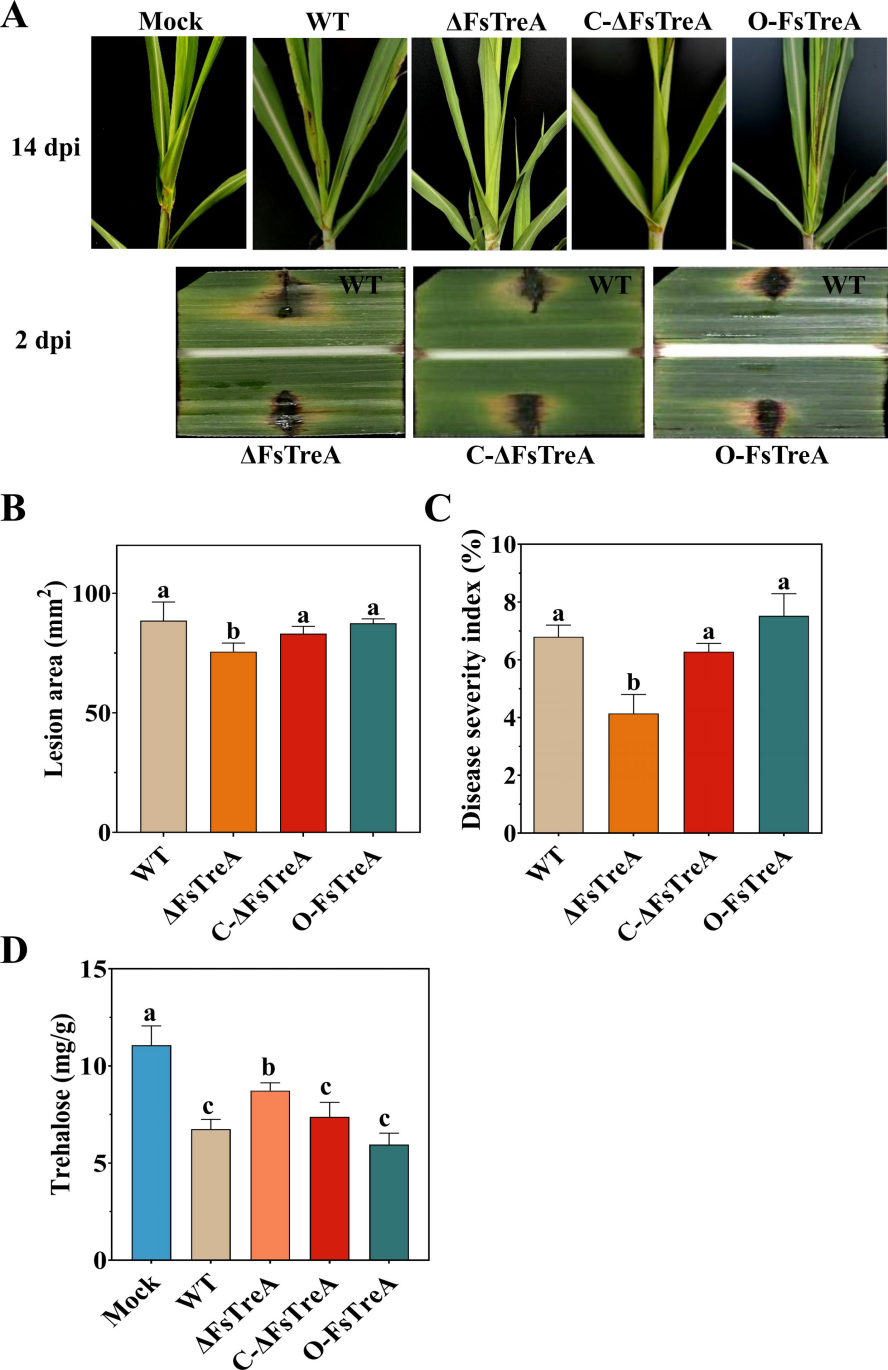
FsTreA mobilizes trehalose from sugarcane tissue, contributing to the virulence of *F. sacchari*. (**A**) Symptoms on sugarcane seedlings and leaves. Photographs were taken at 14 dpi for the plant inoculation assays. For the leaf inoculation assays, representative photographs were taken at 48 h post-inoculation (hpi). The notched leaf margin signifies the inoculation site of WT. (**B and C**) Quantification of disease severity index and lesion area. The disease severity index was determined using 50 seedlings per treatment. Lesion areas of 10 leaves per treatment were measured using ImageJ. (**D**) Trehalose quantification of sugarcane tissues inoculated with *FsTreA* mutant strains. Values represent means ± SE of three biological replicates. Different letters indicate significant differences at *P* < 0.05 as measured by Duncan’s multiple comparisons test.

To assess the impact of FsTreA on the virulence of *F. sacchari*, we inoculated sugarcane plants with conidia from both the wild-type and *FsTreA* mutant strains. Plants inoculated with the Δ*FsTreA* mutant displayed milder PBD symptoms and a lower DSI than those inoculated with WT at 14 dpi. In contrast, plants inoculated with the O-FsTreA mutant showed no significant difference from those inoculated with WT. The virulence of the complementation strain (C-Δ*FsTreA*) was restored by reintroducing a wild-type copy of *FsTreA*. Consistent with *in planta* inoculation assays, the Δ*FsTreA* mutant resulted in significantly smaller lesions on sugarcane leaves compared to the wild type (Fig. 7A through C). These results suggest that FsTreA positively regulates *F. sacchari* virulence.

Since FsTreA functions as an acid trehalase, we propose that its impact on *F. sacchari* virulence is related to the mobilization of extracellular trehalose. To verify this hypothesis, sugarcane tissues inoculated with *FsTreA* mutant strains were analyzed at 48 h post-inoculation. The results indicated significantly higher trehalose accumulation in tissues inoculated with Δ*FsTreA*, followed by those inoculated with the wild type, with the least accumulation observed in tissues inoculated with O-FsTreA (Fig. 7D). We then tested whether adding trehalose to infection sites can rescue the virulence defect of Δ*FsTreA*. The results showed that, compared to the control, exogenous application of trehalose at infection sites increased the DSI across all strains. In particular, the virulence of Δ*FsTreA* was significantly enhanced (Fig. S8). This demonstrates that FsTreA enhances *F. sacchari* virulence by mobilizing host-derived trehalose from sugarcane.

**Fig 8:**
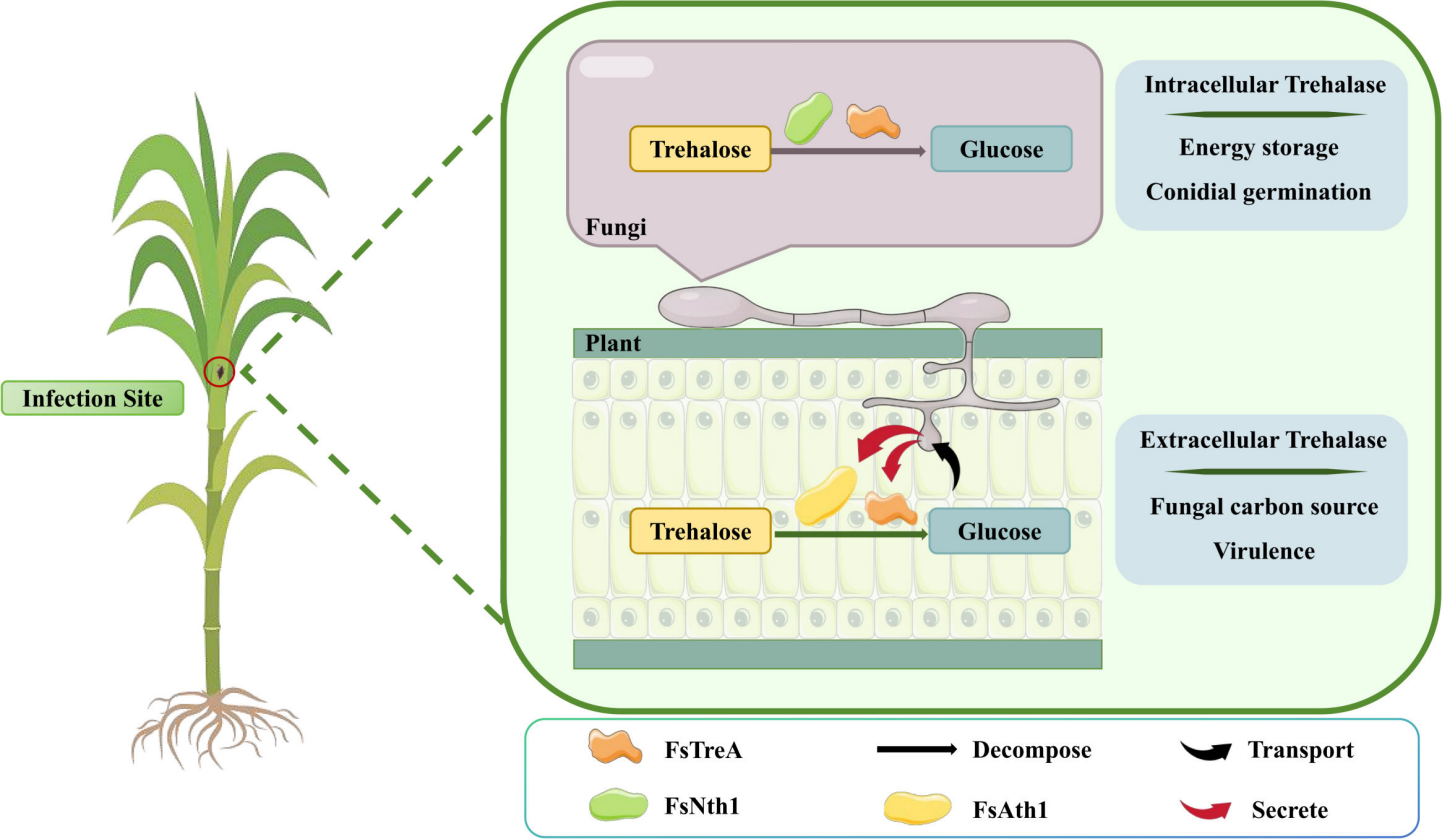
Model of trehalase-mediated dual-compartment trehalose mobilization coordinating development and virulence in *F. sacchari*.

In summary, FsTreA represents an evolutionarily adapted bifunctional trehalase in *F. sacchari*. Intracellularly, it coordinates trehalose mobilization in conjunction with neutral trehalase FsNth1 and acid trehalase FsAth1 through reciprocal compensation, thereby governing conidial germination. During host invasion, the secreted FsTreA utilizes extracellular trehalose from sugarcane as a pathogenic energy source, positively fueling fungal virulence (Fig. 8).

## DISCUSSION

As the primary pathogen responsible for PBD, *F. sacchari* spreads via conidia to establish infection, with conidial germination being crucial for host successful colonization (35). Trehalose is an important reserve carbohydrate that contributes to fungal energy requirements in cell processes such as sporulation, germination, and growth (36). Although trehalases are known to regulate the mobilization of trehalose for conidial germination (37–41), dormancy (42, 43), abiotic stress resilience (44–47), and virulence (48–50), knowledge about trehalases’ function in *F. sacchari* is scarce. Our study identified a unique trehalase FsTreA, distinct from the neutral trehalase FsNth1 and the acidic trehalase FsAth1, which exhibits bifunctional activity: coordinating intracellular trehalose metabolism and utilizing extracellular trehalose from sugarcane as an energy source to sustain virulence.

Trehalases facilitate fungal metabolism by hydrolyzing trehalose into two glucose molecules. Fungal trehalases are traditionally classified into neutral (cytosolic, GH37 family) and acidic (vacuolar/cell surface-localized, GH65 family) types (51, 52). Neutral trehalases like *S. cerevisiae* Nth1 possess a Ca²^+^- binding domain (CaBD) and conserved domain (CD), while acidic trehalases, Ath1, only have a CD. Additionally, acid trehalases in pathogenic fungi possess an N-terminal signal peptide that directs their extracellular secretion, facilitating the utilization of exogenous trehalose as a carbon source, such as TRE1 in *Magnaporthe grisea* and *Gibberella zeae* (21, 53). In this work, we classified the classic types of trehalases in *F. sacchari* as the acid and neutral trehalases, as FsAth1 and FsNth1, respectively, assigning them into the GH65 and GH37 glycoside hydrolase families. Unexpectedly, a specific trehalase, FsTreA, represents a novel variant: it belongs to the GH37 family, but lacks the CaBD characteristic of neutral trehalases yet contains an SP and a trehalase domain (Fig. 1). Structurally, the coexistence of a GH37 catalytic domain and signal peptide in FsTreA provides a structural basis for its dual localization and function—enabling intracellular metabolic coordination and extracellular nutrient acquisition. This structure challenges the traditional dichotomous classification of fungal trehalases, indicating that *F. sacchari* has evolved a multifunctional trehalase to adapt to its pathogenic lifestyle. To elucidate its functional nature, we characterized the enzymatic properties of FsTreA. The pH profile revealed a broad pH range (pH 1.0–12.0) for FsTreA, peaking under acidic conditions (pH 3.0–4.0) while maintaining moderate activity under neutral conditions (pH 6.0–7.0), albeit lower than under acidic conditions. This pattern is consistent with its bifunctional nature (Fig. S10A). Notably, its enzymatic activity was independent of Ca²^+^, distinguishing it from conventional neutral trehalases (Fig. S10C). Furthermore, substrate specificity analysis confirmed that FsTreA exhibits high specificity for trehalose, supporting its role as a dedicated hydrolase (Fig. S10B). Together, these findings illustrate how *F. sacchari* has evolved this multifunctional trehalase to adapt to its pathogenic lifestyle, integrating both structural innovation and biochemical versatility.

To further investigate the role of FsTreA in *F. sacchari*, we analyzed its expression pattern during conidial germination and hyphal morphogenesis. Unlike *FsAth1*, both *FsTreA* and *FsNth1* were significantly upregulated during this process, exhibiting peak expression at 6 h, with the transcript level of *FsTreA remaining* higher than that of *FsNth1* (Fig. 2). The results indicate that *FsTreA* and *FsNth1* contribute to the germination and growth of *F. sacch*ari. Then we engineered knockout (Δ*FsTreA*, ΔFsNth1, and ΔFsAth1), complementation (C-Δ*FsTreA*), and overexpression (O-FsTreA) mutant strains (Fig. S4 and S5). The significant depletion of trehalose in O-FsTreA spores indicates that FsTreA plays a role in mobilizing intracellular trehalose, similar to neutral trehalase in various fungi (Fig. 5D) (54, 55). In *Aspergillus niger*, deleting the intracellular trehalase gene *treB* resulted in fewer and less viable spores, and during the early stages of conidial germination, the internal levels of trehalose were higher than in the wild type (56). These findings were similar to those of ΔFsNth1. Deletion of *FsNth1* resulted in severely impaired growth and development and reduced the germination rate to one-third of that in WT (Fig. S11). However, the disruption of *FsTreA* markedly accelerated trehalose mobilization and enhanced germination and conidiation (Fig. 4D). The results were contradictory to the expected phenotype of impaired trehalose mobilization. To explain this counterintuitive result, the expression of *FsNth1* and *FsAth1* in Δ*FsTreA* was quantified. The results showed that the expression of *FsNth1* and *FsAth1* in Δ*FsTreA* was significantly upregulated compared to WT. Conversely, the overexpression of *FsTreA* almost entirely suppressed *FsNth1* expression (Fig. 5B). These results suggest that FsTreA and FsNth1 collaboratively regulate intracellular trehalose mobilization in *F. sacchari* via a reciprocal compensatory mechanism. This mechanism serves as a key adaptive trait for the fungus and provides an important insight into the regulation of fungal carbon metabolism.

Subsequently, we verified the ability of FsTreA to utilize extracellular trehalose. The yeast signal sequence trap and subcellular localization assay revealed that FsTreA can be secreted extracellularly and primarily localized to the cell surface (Fig. 6A through C), while enzymatic assays showed reduced extracellular trehalase activity in Δ*FsTreA* and elevated activity in O-FsTreA (Fig. 6E). In conclusion, these results demonstrate for the first time that FsTreA functions as an acid trehalase capable of mobilizing extracellular trehalose, which is a critical trait for pathogens to acquire host nutrients. Intriguingly, emerging evidence suggests that trehalose from hosts acts as an important carbon source for many pathogens and can support their growth and colonization (57). Despite the availability of exhaustive evidence documenting the biological benefits of trehalose in microbial pathogens, the precise mechanism by which host trehalose could be acquired by the filamentous fungi remains unknown. Critically, trehalase activity demonstrates a dose-dependent enhancement of virulence in FsTreA mutants, indicating that *F. sacchari* hijacks sugarcane trehalose to boost its infectious capacity, mirroring the carbon piracy strategy reported in *Phytophthora sojae* (58).

Notably, O-FsTreA did not show enhanced virulence (Fig. 7). We propose three hypotheses. First, secretory bottleneck: FsTreA secretion may reach saturation in the host; overexpression fails to enhance extracellular enzyme activity. For example, ER stress in rice downregulates PR proteins via *OsIRE1* to reduce secretory load and avoid overload (59). Second, feedback inhibition: accumulated glucose from trehalose hydrolysis may trigger carbon metabolic feedback, such as glucose accumulation activating *CCR* in *M. oryzae*, thereby impairing the pathogen’s capacity to develop infective structures like appressoria and invade host plants (60). Third, host immune response: excess FsTreA may be recognized by sugarcane. Just as trehalase peptides from nematodes can activate the host MAPK response, FsTreA is likely to induce this MAPK-mediated response or other defense mechanisms, thereby offsetting its carbon acquisition advantage (61). Further experiments—such as constructing FsTreA mutants with varying secretion efficiencies or detecting host defense gene expression—are needed to validate these hypotheses.

In summary, our results further reveal that FsTreA is a bifunctional trehalase in filamentous fungi: beyond its acid trehalase activity for extracellular trehalose mobilization, it also orchestrates intracellular trehalose metabolism in concert with FsNth1 and FsAth1. Together, these roles allow FsTreA to potentiate *F. sacchari* virulence. It provides comprehensive insight into the function of trehalase and mechanisms in filamentous fungi and expands understanding of pathogen-host nutrient competition. Furthermore, FsTreA makes it a potential target for PBD control due to its dual role in metabolism and its role in virulence, advancing the development of novel antifungal strategies. Our work enriches the understanding of pathogen-host nutrient competition and lays the foundation for further mechanistic studies on fungal pathogenicity.

## Data Availability

The genome sequences of FsTreA, FsNth1, and FsAth1 are available in the NCBI database under accession no. PX557837, PX557838, and PX557839, respectively.

## SUPPLEMENTAL MATERIAL

Text S1 aem.00697-26-s0001.docx Sequence alignment.

Supplemental figures aem.00697-26-s0002.docx Fig. S1 to S11.

Table S1 aem.00697-26-s0003.docx Primers used in this study.

ASM does not own the copyrights to Supplemental Material that may be linked to, or accessed through, an article. The authors have granted ASM a non-exclusive, world-wide license to publish the Supplemental Material files. Please contact the corresponding author directly for reuse.
