# Supplementary material for: Bifunctional trehalase FsTreA coordinates intracellular mobilization and extracellular utilization of trehalose to modulate virulence in Fusarium sacchari
Source: Appl Environ Microbiol. 2026 May 14;92(6):e00697-26. doi: 10.1128/aem.00697-26 (PMC13274424; doi:10.1128/aem.00697-26)
Supplement: Text S1 — Sequence alignment. [file aem.00697-26-s0001.docx]

**Bifunctional Trehalase FsTreA Coordinates Intracellular Mobilization and Extracellular Utilization of Trehalose to Modulate Virulence in *Fusarium sacchari***

**Yuejia Chen^1†^, Yueying Zhao^1†^, Gengzhong Cheng^1^, Yuming Lin^1^, Qianmin Liang^1^, Lianke Zhu^1^, Liuting Huang^1^, Xinyi Lin^1^, Ziting Yao^2*^, Chengwu Zou^1*^**

^1^ State Key Laboratory for Conservation and Utilization of Subtropical Agro-Bioresources, Ministry & Province Sponsored Center of Collaborative Innovation for Sugarcane Industry, College of Agriculture, Guangxi University, Nanning 530004, China;

^2^ Plant Protection Research Institute, Guangxi Academy of Agriculture Science, Nanning 530007, China

^*^Correspondence: yaoziting@gxaas.net(Z.Y.); zouchengwu@gxu.edu.cn (C.Z.)

^†^ Co-authors: 18172258103@163.com (Y.C.); zhaoyy13317799307@163.com(Y.Z.)

**Abstract**

*Fusarium sacchari* causes devastating Sugarcane Pokkah Boeng Disease (PBD) by infecting tissues through conidia that enter via micro-wounds or stomata on the leaf surface, making conidial germination a critical step in its pathogenicity. Here, we identified a trehalase, FsTreA, which contains not only the essential trehalase structural domain characteristic of neutral trehalases but also features an N-terminal signal peptide. *FsTreA* exhibits the highest transcriptional level among all trehalases during conidial germination. Disruption of *FsTreA* significantly elevated intracellular trehalose accumulation in conidia, confirming its role in intracellular mobilization. However, ΔFsTreA showed accelerated trehalose mobilization rates during germination, coinciding with enhanced conidial germination and increased conidial yield. This was attributed to functional compensation by two other trehalases, FsNth1 and FsAth1. Furthermore, we found that FsTreA predominantly localizes to the cell surface and possesses the ability to mobilize extracellular trehalose. Moreover, the virulence of *FsTreA* mutant strain was positively correlated with the capability for extracellular trehalose mobilization. In conclusion, our results demonstrate that the bifunctional trehalase FsTreA coordinates intracellular and extracellular trehalose utilization to modulate virulence in *F. sacchari*.

**IMPORTANCE**

Sugarcane Pokkah Boeng Disease (PBD), caused by *Fusarium sacchari*, poses a severe threat to global sugarcane production. This study represents the first identification of the bifunctional trehalase FsTreA in *F. sacchari*, which breaks the traditional dichotomous classification framework of traditional fungal trehalases. FsTreA harbors both the GH37 catalytic domain characteristic of neutral trehalases and an N-terminal signal peptide conferring secretory trait of acid trehalases. This enzyme participates in intracellular trehalose mobilization and is simultaneously secreted extracellularly to hijack host-derived trehalose as a carbon source, constituting a distinctive infection strategy that provides new insights into host-pathogen interaction mechanisms. These findings advances the theoretical understanding of fungal metabolic regulation, holding significant scientific and practical value for safeguarding the security of the global sugarcane industry.

***Keywords****:* Sugarcane; Pokkah Boeng Disease; *Fusarium sacchari;* Bifunctional trehalase; Virulence

1. **Introduction**

Sugarcane (*Saccharum spp.*) is a fundamental raw material for sugar production^[1]^. However, the sugarcane industry has long been threatened by various diseases.PBD, a prominent epidemic fungal disease, significantly reduces both the yield and sucrose content of sugarcane^[2-4]^. To date, studies on PBD have predominantly focused on pathogen identification, germplasm resistance, and the impacts of environmental factors on disease progression^[5-7]^. Nevertheless, identifying the causal agent and delineating its pathogenesis remain strategic imperatives for effective PBD control.

*F. sacchari* is the primary pathogen responsible for PBD in China, invading the host through micro-wounds via conidia to establish infection. Consequently, conidial germination constitutes the cardinal determinant of successful host colonization. Trehalose is a stable non-reducing disaccharide composed of two glucose units linked in an α,α-1,1 configuration^[8]^. It is widely present in microorganisms such as bacteria and fungi^[9, 10]^. In fungi, trehalose serves not only a key energy-storage compound (accounting for up to 15% in spores)^[11, 12]^, but also provides a carbon source for germination^[13]^. Additionally, it helps spores resist environmental stress prior to germination. In yeast, trehalose in spores is essential for survival to long-term desiccation^[14]^. In arbuscular mycorrhizal fungi, trehalose aids recovery in response to heat shock and arsenate stress^[15]^. Fungi urgently need to mobilize trehalose during the critical process of conidial germination^[16]^.

Trehalose mobilization depends on trehalases^[17]^. These enzymes are crucial for fungal carbon source metabolism, stress adaptation, and pathogenic processes^[18, 19]^. Currently, research on genes encoding trehalases has established a substantial foundation. They also play important roles in metabolic regulation. In *Saccharomyces cerevisiae*, dual deletion of *NTH1* and *ATH1* elevates intracellular trehalose concentrations while impairing conidial germination and carbon utilization^[20]^. The rice blast pathogen *Magnaporthe oryzae* requires *NTH1* for host colonization; deletion mutants exhibit attenuated pathogenicity^[21]^. In *Candida albicans,* the gene encoding the cell wall-associated acid trehalase, *Atc1*, has emerged as a promising therapeutic target for novel antifungal agents. The gene-knockout mutant exhibited impaired hyphal and pseudohyphal formation capabilities, which consequently led to a significant reduction in its pathogenicity^[22, 23]^. However, the regulatory mechanism of trehalose metabolism, especially the role of trehalase, remains unclear in *F. sacchari*.

In the present study, we identified a bifunctional trehalase, FsTreA, which coordinates trehalose mobilization in conjunction with neutral trehalase FsNth1 and acid trehalase FsAth1 through reciprocal compensation, thereby regulating germination and sporulation. Additionally, FsTreA utilizes extracellular trehalose from sugarcane as an energy source, thereby enhancing fungal virulence. These findings demonstrate that trehalase is essential for the development and virulence of *F. sacchari* through trehalose mobilization.

1. **Materials and methods**

*2.1 Strains and culture conditions*

The wild-type strain CNO-1 of *F. sacchari* was used for the construction of *FsTreA* mutant strains^[24]^. All strains were grown at 28 ℃ on potato dextrose agar (PDA) plates for 7 days to assess their phenotypic traits. Sporulation was measured as described previously^[25]^. For conidial germination assays, spores were harvested from 7-days-old colonies on PDA plates and then inoculated in PDB liquid medium at 28 ℃ with shaking at 150 rpm for 6 hours. The *Escherichia coli* strains DH5α and BL21(DE3) were used for plasmid construction and expressing trehalases, respectively. They were cultured on lysogeny broth medium at 37℃.

*2.2 Bioinformatic analysis*

Signal peptides were identified by the SignalP 5.0 server (https://services.healthtech.dtu.dk/services/SignalP-5.0/). Protein domains were predicted by Pfam (https://pfam.xfam.org/). The homologous sequences were compared using BLASTp in the NCBI database, and sequences with high similarity were selected. Phylogenetic tree was constructed using MEGA 7 with the Maximum Likelihood method, and iTOL v7 for visualization(https://itol.embl.de/) ^[26]^.

*2.3 RNA extraction and quantitative real-time reverse transcription PCR*

The total RNA from each strain was extracted using the RNA extraction kit (TaKaRa, Beijing, China) according to the manufacturer's protocol. First-strand cDNA synthesis was performed using the FastQuant RT Kit (TaKaRa, Beijing, China). Quantitative real-time PCR was conducted using the SuperReal PreMix Plus (TaKaRa, Beijing, China) with the target gene primer pairs (Table S1) and 18S rRNA as an internal control^[27]^. The relative expression levels were calculated using the 2^−∆∆Ct^ method^[28]^. The data are presented as the mean ± SE from three independent biological replicates.

*2.4 Trehalase encoding genes related mutant strains construction*

To knock out *FsTreA*, *FsNth1* and *FsAth1*, deletion fragments were amplified using fusion PCR with the primer pairs FsTreA-A/B, FsNth1-A/B and FsAth1-A/B, respectively (Table S1). The hygromycin resistance gene (*Hph*) was employed as selection marker^[29]^. The amplified fragments were transformed into protoplasts of the wild-type strain CNO-1 using a polyethylene glycol (PEG, MW 3350, 40% w/v)-mediated method^[30]^, resulting in the trehalase encoding genes deletion mutants, named ΔFsTreA, ΔFsNth1 and ΔFsAth1, respectively. For the complementation strains, full-length *FsTreA* fragments, including promoter sequences, were amplified using the primer pair C-FsTreA-F/C-FsTreA-R (Table S1). These fragments were cloned into the pCPXG418 vector using the pEASY-Basic Seamless Cloning and Assembly Kit (Transgen Biotech, Beijing, China) and then transformed into the protoplasts of Δ*FsTreA*. The overexpression strains were developed by introducing additional copies of *FsTreA* into the CNO-1 protoplasts, which were then selected on media supplemented with the appropriate antibiotics.

*2.5 Trehalose content and trehalase activity quantification*

For the detection of trehalase activity in fungal strains, 1 mL of the spore suspension (1×10^7^ conidia/mL) of each strain was inoculated into 100 mL PDB liquid medium and cultured for 3 days^[31]^. Mycelia were filtered through four layers of gauze, and the remaining culture medium was centrifuged to collect spores for testing. For the detection of trehalose content in the host tissues, 1 cm of tissue around the inoculation site was collected, ground in liquid nitrogen, and then tested. The trehalase activity and trehalose content were measured using the Solarbio (Beijing, China) Trehalase (THL) Activity Assay Kit and Trehalose Content Assay Kit, respectively, following the instructions provided with each kit.

*2.6 Yeast signal sequence trap experiment*

Functional validation of the predicted signal protein was conducted with a yeast secretion system^[32]^. DNA fragments encoding SP of FsTreA were amplified using primers pSUC2-SP^FsTreA^-F/R and introduced into pSUC2 at the N-terminal of the invertase. The pSUC2-SP^FsTreA^ vector was transformed into the yeast strain YTK12 and screened on CMD-W medium. Positive colonies were replica-plated on YPRAA medium plates to detect invertase secretion and subjected to the TTC colour reaction assay. YTK12 transformed with pSUC2-SP^Avr1b^ and the empty pSUC2 vector was used as a positive and negative control, respectively.

*2.7 Subcellular localization analysis*

To construct the plasmids of FsTreA - mCherry, the full - length of *FsTreA* was amplified and inserted into pCPXG418 - mCherry digested by *Not* Ⅰ. Then, it was transformed into CNO - 1, resulting in an FsTreA fluorescence - labeled strain, named FsTreA::mCherry^[33]^. To confirm the expression of the mCherry fusion proteins, hyphae were cultured on PDA for 48 hours, and mCherry signals were observed and photographed using an Olympus DP70 microscope (Olympus, Tokyo, Japan). To further vertify the location of FsTreA, the cell wall and cytoplasmic proteins of FsTreA::mCherry were extracted using protein Extract Kit (Beyotime Biotech, ShangHai, China). Western blotting was performed on the cell wall and cytoplasmic frafctions of each strain using Anti-mCherry (Sanying Biotech, WuHan, China).

*2.8 Virulence assay*

To assess the virulence of FsTreA mutants, sugarcane plants at the 5-leave stage were inoculated with a 300 µL volume of conidial suspensions (1×10^4^ conidial/mL) from each strain. Disease severity was evaluated 14 days post-inoculation (dpi), and the disease severity index (DSI) was calculated using a symptom severity scale^[25]^. The DSI was determined using the formula DSI = 100 × (Σ score/5N), where N represents the number of observed seedlings (N = 50). Each assay was replicated three times. Sugarcane leaves were scraped with a blade and then inoculated with mycelial plugs (6 mm in diameter). The leaves were then incubated at 28°C^[34]^. The amount of leaf necrosis was observed and photographed after 2 days. Disease assays on transgenic sugarcane plants were conducted using the methodology described previously.

*2.9 Expression and purification of trehalases*

FsNth1, FsTreA, and FsAth1 (without SP) were amplified and cloned into pGEX - GT digested by *EcoR* Ⅰ and *BamH* Ⅰ, respectively. Three trehalase recombinant proteins were expressed in *E. coli* strain BL21(DE3). Expression was induced by adding 0.2 mM IPTG for 12 hours at 16°C. For protein extraction, supernatant was collected by centrifugation at 5000 rpm for 30 min. trehalases were purified using GST resin (Beyotime Biotech, ShangHai, China) following the manufacturer’s instructions. The purification protein samples were concentrated using centrifugal filter devices with 50 KDa (Sangon Biotech, Shanghai, China). Recombinant protein expression was verified by concentration determination using the BCA method and by Western Blot analysis using the anti-GST antibody (Abcam, Shanghai, China).

*2.10 Statistical analysis*

GraphPad Prism 9.0 was used for the statistical analysis of the numerical data. Significant differences were analysed using two-tailed Student’s t-test or one-way analysis of variance (ANOVA) followed by Duncan’s multiple comparisons test. Asterisks are used to indicate *p* < 0.05 (*), *p* < 0.01 (**), and *p* < 0.001 (***). Different letters indicate significant differences at *p* < 0.05.

1. **Results**

*3.1 Identification of trehalases in F. sacchari*

Trehalose mobilization serves as a critical energy source to power conidial germination. To identify trehalases in *F. sacchari*, amino acid sequences of Nth1 and Ath1 from *S. cerevisiae* were used as queries in BLAST searches against the genome of *F. sacchari* strain CNO-1. *FsAth1* (PX557839) and *FsNth1* (PX557838) were identified, with similarities of 49.7% and 47.9% respectively. Notably, we discovered a novel trehalase, FsTreA (PX557837), which contains not only the essential trehalase domain characteristic of neutral trehalases but also possesses an N-terminal signal peptide specific to acid trehalases (Figure 1A). Signal peptide prediction using SignalP 5.0 revealed that FsTreA possesses a 20-amino acid signal peptide with a predicted cleavage site between Ala20 and Leu21. Compared to other trehalases, FsTreA exhibits the lowest sequence similarity to *S. cerevisiae* Nth1 and Ath1, with merely 30.3% and 20.9% identity, respectively. Phylogenetic analysis confirmed that FsTreA constitutes a distinct clade, separate from both neutral and acid trehalases. Despite the low similarity, FsTreA and FsNth1 are both members of the GH37 glycoside hydrolase family. FsTreA is phylogenetically more closely related to neutral trehalases than to acidic trehalases (Figure 1B), suggesting functional conservation within the neutral enzyme lineage.


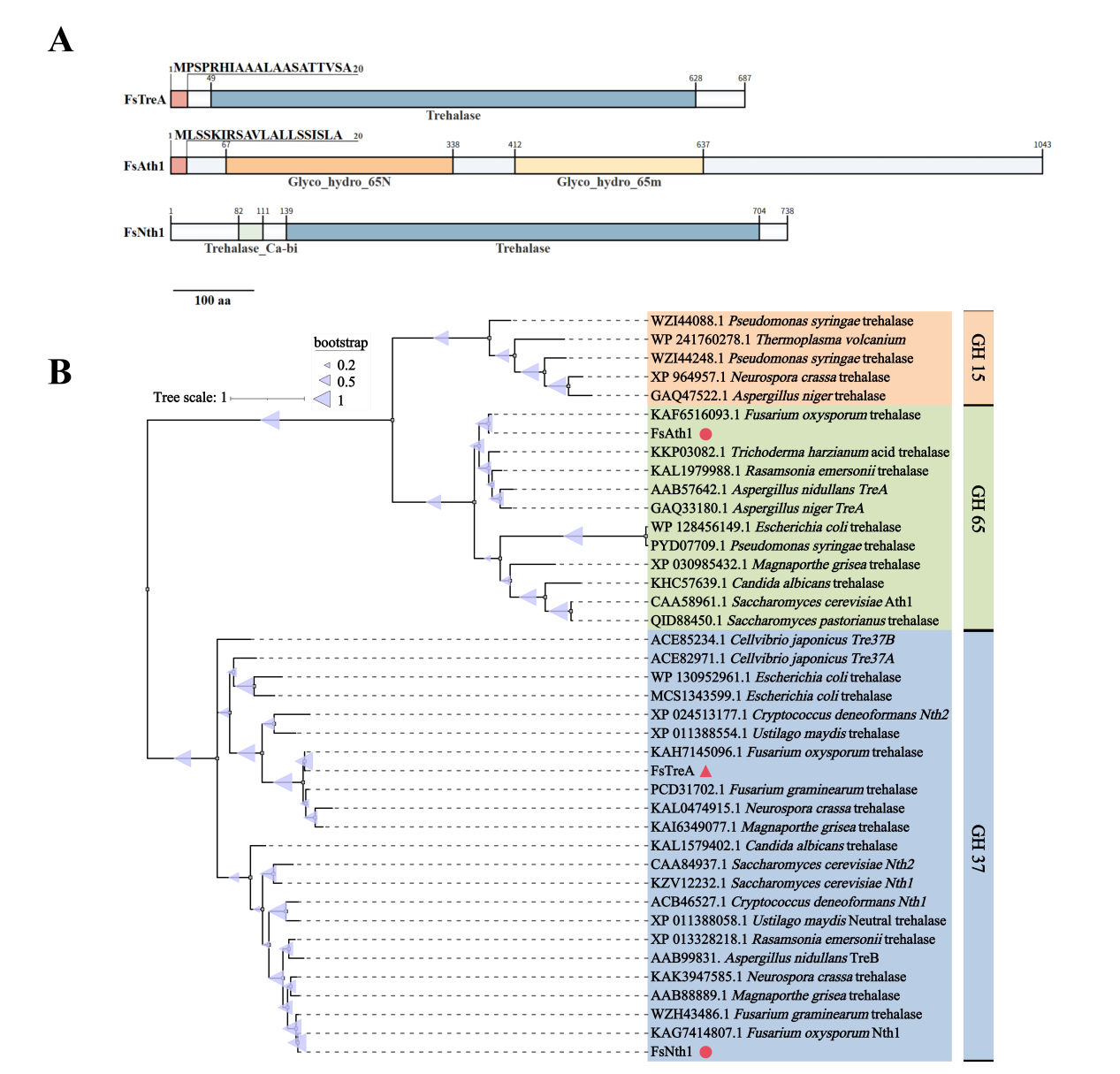


**Fig. 1 FsTreA, FsAth1, and FsNth1 are trehalases.** (A) Prediction of the signal peptide and domain architecture for the three trehalases in *F. sacchari*. (B) Phylogenetic tree of the three trehalases constructed using the Maximum Likelihood method with MEGA 7, Colored stripes represent the protein family to which the corresponding sequences belong.

*3.2 FsTreA exhibits the highest expression compared to other trehalases during conidia germination*

To investigate the roles of these three trehalases in *F. sacchari*, we examined the transcriptional levels of trehalases across different stages of conidial germination, obtaining expression profiles of *FsTreA*, *FsAth1,* and *FsNth1* during conidial germination (6 hours) and hyphal morphogenesis (8 and 10 hours) (Figure 2A). Transcriptomic profiling revealed that *FsTreA* and *FsNth1* were significantly upregulated compared to *FsAth1* during this process. *FsTreA* exhibited peak expression at 6 hours, at levels 1.5-fold higher than *FsNth1*, whereas its expression stabilized during hyphal formation. In contrast, *FsAth1* expression remained unchanged throughout (Figure 2B). These findings suggest that when genes encoding all three trehalases coexist, *FsTreA* and *FsNth1* coordinately regulate the development of *F. sacchari*, with *FsTreA* serving as the primary regulator during conidial germination.


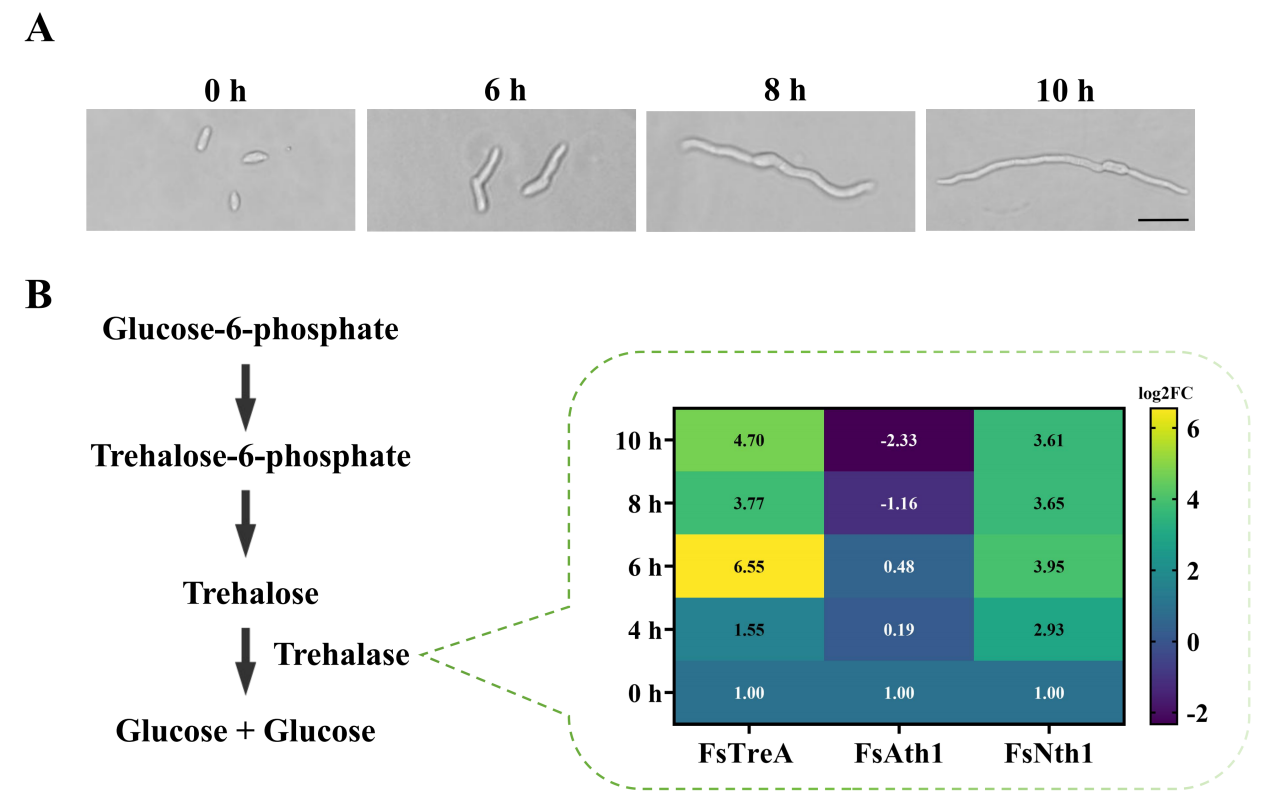


**Fig. 2 *FsTreA* exhibits the highest expression during conidial germination.** (A) Development of conidial germination and hyphae. The spores of CNO-1 were inoculated in PDB medium, and representative pictures were captured at 0 h, 6 h, 8 h and 10 h. Scale bar = 25 µm. (B) Relative expression of *FsTreA*, *FsAth1* and *FsNth1* during conidial germination and hyphal morphogenesis. The transcript level of conidia was set to a value of 1.0, yellow indicates high expression, purple indicates low expression.

*3.3 FsTreA regulates F. sacchari sporulation and germination*

To investigate the biological role of *FsTreA* in *F. sacchari,* we generated the *FsTreA* deletion mutant Δ*FsTreA* by replacement with the hygromycin resistance gene (Figure 3 & Figure S3). The reintroduction of the wild-type copy of *FsTreA* into Δ*FsTreA* constructed the complementation strain, designated C-Δ*FsTreA*, and the *FsTreA* overexpression mutant strain, designated O-*FsTreA* (Figure S1 & Figure S2). The wild-type strain CNO-1, Δ*FsTreA*, O-FsTreA and C-Δ*FsTreA* were used for phenotypic analyses.


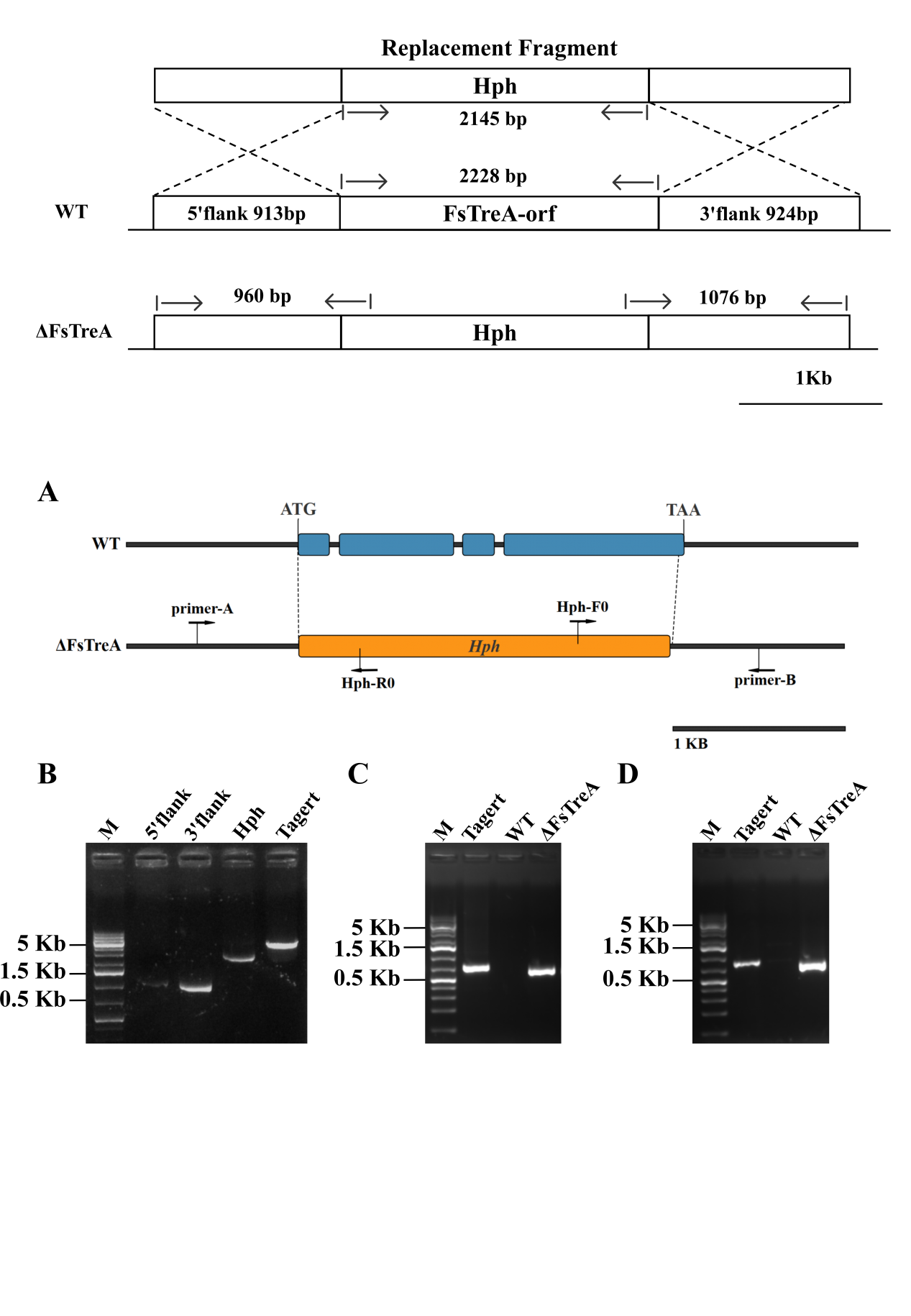


**Fig. 3 Generation of *FsTreA* deletion mutant strains.** (A, B) *FsTreA* gene locus and gene replacement construct. (C, D) *FsTreA* deletion mutants were validated by PCR with primer A / Hph-R0 and Hph-F0 / primer B, respectively.

Phenotypic traits indicated that the deletion of *FsTreA* did not affect the growth of *F. sacchari,* but significantly increased conidial yield and germination rate by 36% and 44%, respectively, compared to the wild-type. Conversely, the overexpression of *FsTreA* enhanced the development of *F. sacchari*, increasing colony diameter by 8.7% compared to the wild-type. Complementation of *FsTreA* restored phenotypes to wild-type levels without any statistically significant difference (Figure 4). These results suggest that *FsTreA* modulates sporulation and germination in *F. sacchari*.


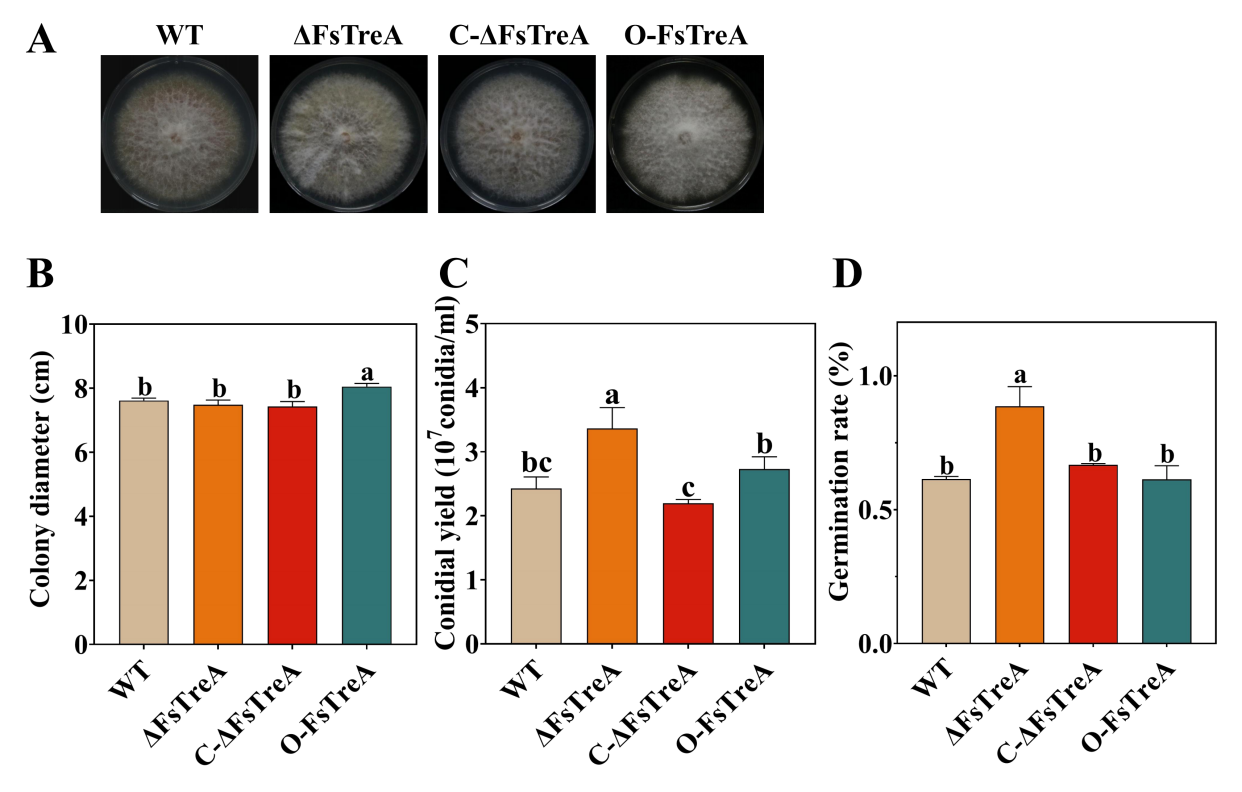


**Fig. 4 *FsTreA* regulates sporulation and germination in *F. sacchari.*** (A) Phenotypes of *FsTreA*-related mutants. All strains were inoculated on PDA plates at 28 ℃ for 7 days. (B) Colony diameters of all strains were measured. (C) Statistics of conidial yield for all strains. Conidia were harvested from 7-day-old colonies on PDA plates. (D) Conidial germination rates in PDB at 28 ℃ for 6 hours. All experiments were replicated three times. Values represent means ± SE of three biological replicates. Different letters indicate significant differences at *p* < 0.05 as measured by Duncan’s multiple comparisons test.

*3.4 The mobilization of intracellular trehalose is coordinately regulated by FsTreA, FsNth1, and FsAth1 through a reciprocal compensatory mechanism*

Fungi require trehalases to mobilize intracellular trehalose for conidial germination. However, our results demonstrate that the deletion of *FsTreA* enhances both germination and conidiation, as shown in Figure 4. To investigate this phenomenon, we measured the trehalose mobilization capacity and intracellular content of trehalose in *FsTreA*-related mutants during conidial germination and hyphal morphogenesis. These data reveal that overexpression of *FsTreA* significantly accelerates intracellular trehalose consumption in *F. sacchari*, while the trehalose accumulation in Δ*FsTreA* conidia is substantially higher than that in WT conidia, thus conclusively establishing that FsTreA plays an essential role in trehalose mobilization (Figure 5A, C). However, Δ*FsTreA* exhibited faster trehalose mobilization rates than WT (Figure 5A, D), with the trehalose accumulation levels in the hyphae showing a non-significant difference. Since *F. sacchari* harbors three trehalase isozymes, this accelerated mobilization phenotype likely points to functional compensation by the remaining trehalases (FsAth1 and FsNth1) following *FsTreA* deletion. To verify this hypothesis, we quantified the trehalase genes expression levels of Δ*FsTreA* and O-FsTreA. The disruption of *FsTreA* significantly increased the expression of *FsAth1* and *FsNth1* to 42-fold and 34-fold of the wild-type levels, respectively (Figure 5B). In contrast, the overexpression of *FsTreA* sustained the induction of *FsAth1* but nearly eliminated FsNth1 expression. These findings indicate that FsTreA, FsNth1, and FsAth1 cooperatively regulate intracellular trehalose mobilization in *F. sacchari* through a reciprocal compensatory manner.


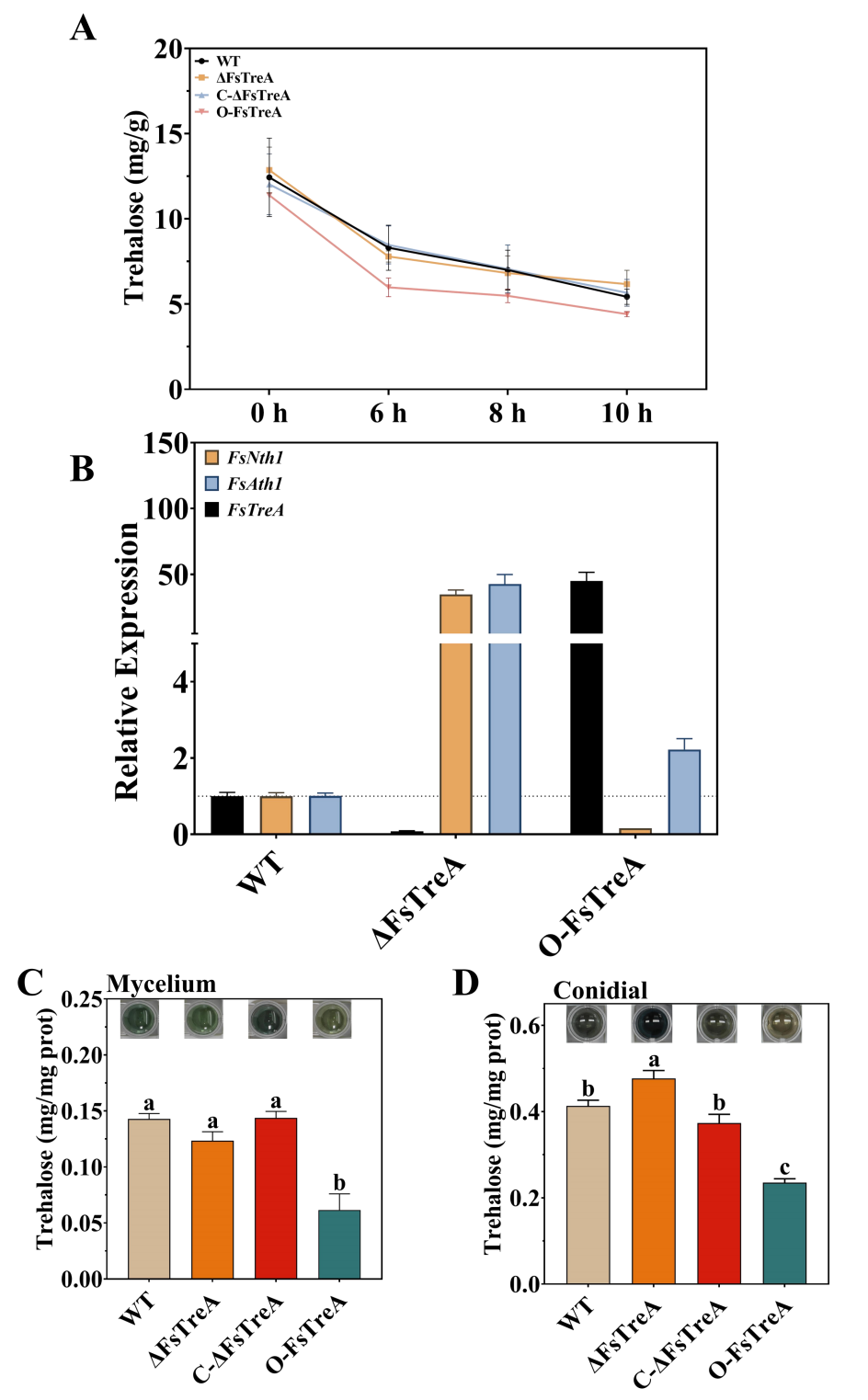


**Fig. 5** ***FsTreA*, *FsNth1*, and *FsAth1* cooperatively regulate intracellular trehalose mobilization in *F. sacchari.*** (A) Trehalose quantification content in *FsTreA* mutants across developmental stages of germination and hyphae (on a fresh weight basis). (B) Expression patterns of three genes encoding trehalases in *FsTreA* mutants. The expression of three genes was measured by RT-qPCR (2^-ΔΔct^ method) with 18S rRNA as an internal reference. Transcript levels of the three genes in the wild-type were normalized to 1.0. (C) Trehalose quantification in mycelium of *FsTreA* mutants. (D) Trehalose quantification in conidia of *FsTreA* mutants. Values represent means ± SE of three biological replicates. Different letters indicate significant differences at *p* < 0.05 as measured by Duncan’s multiple comparisons test.

*3.5 FsTreA exhibits secretory activity similar to acid trehalases*

Similar to FsAth1, FsTreA possesses an N-terminal signal peptide characteristic of acid trehalases. To validate the secretory capacity of FsTreA, a yeast signal trap assay was performed. The coding sequence of the N-terminal region of FsTreA (MPSPRHIAALAASATTVSA) was cloned into the yeast invertase vector pSUC2, and then all the constructs were transformed into the yeast strain YTK12. The strain containing *PsAvr1b* was used as the positive control in this assay. Only constructs containing the fused FsTreA and PsAvr1b could grow on YPRAA medium and catalyze the conversion of 2,3,5-triphenyltetrazolium chloride (TTC) to the red product triphenylformazan (Figure 6B). In contrast, YTK12 and the strain carrying the pSUC2 vector used as a negative control did not change the color of the culture. These results confirmed that FsTreA exhibits secretory activity.

Furthermore, we determined the subcellular localization of FsTreA. As expected, the red fluorescence signal of the FsTreA-mCherry strain was predominantly observed at the cell surface (Figure 6C & Figure S6). Subsequently, we inoculated FsTreA mutants on MM plates with trehalose as the sole carbon source. The colony diameter of O-FsTreA was significantly increased, but Δ*FsTreA* showed no difference compared to WT. However, when cultured for 7 days, the growth of the Δ*FsTreA* was significantly inhibited compared to WT (Figure 6D & Figure S7A). Additionally, we performed enzymatic assays on secreted protein, which revealed significantly reduced trehalase activity in Δ*FsTreA* mutants, while O-*FsTreA* exhibited markedly higher activity than WT (Figure 6E). This demonstrates that FsTreA functions as a secreted acid trehalase capable of mobilizing extracellular trehalose.


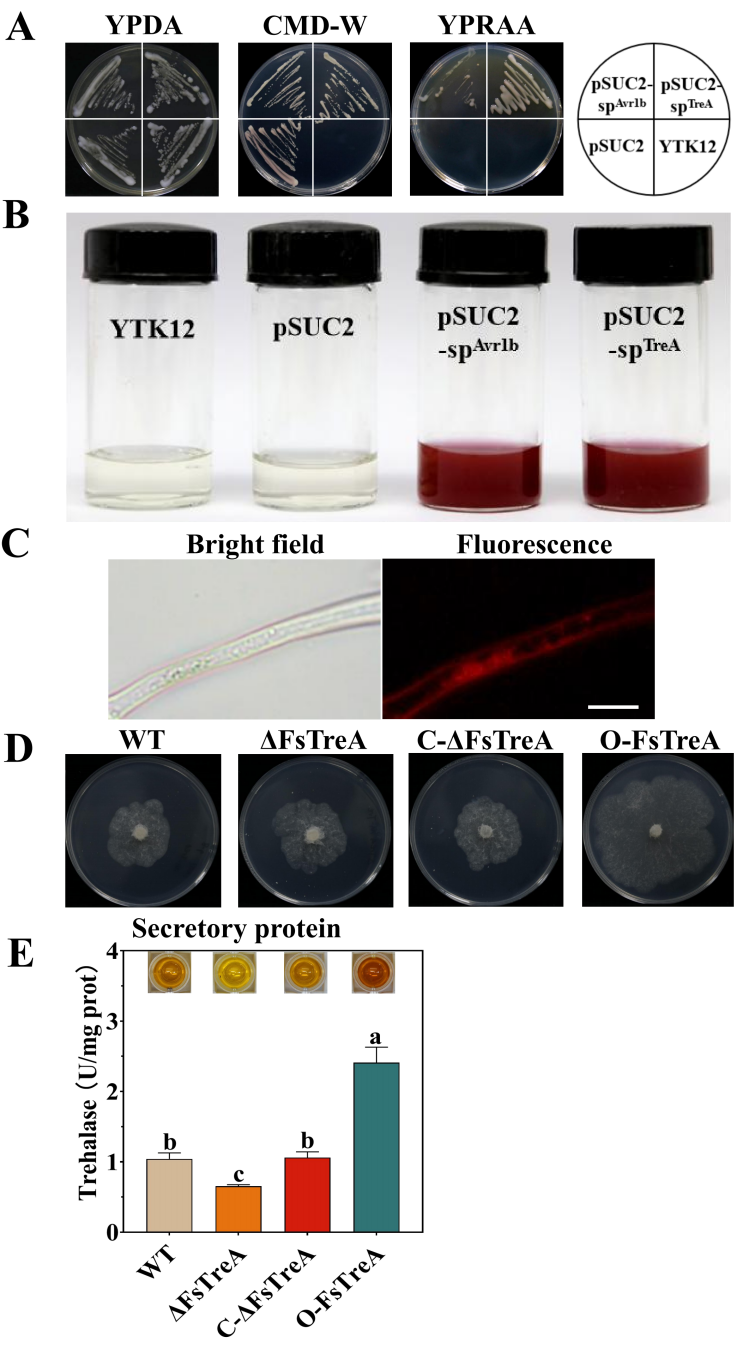


**Fig. 6** **FsTreA demonstrates functional attributes of an acid trehalase in *F. sacchari*.** (A) Validation of the N- terminal signal peptide of FsTreA by yeast secretion system. The SP was fused to mature yeast invertase. The YTK12 strain is confined to YPDA medium for growth. Those with the SP-fused vector thrive on YPDA, CMD-W, and YPRAA media. (B) Secreted invertase can reduce triphenyltetrazolium chloride (TTC) to red formazan. The pSUC2-sp^Avr1b^ was used as the positive control, and the empty pSUC2 vector as the negative control, both cultured on YPDA plates for 2 days. (C) Subcellular location of FsTreA in *F. sacchari*. FsTreA::mCherry was constructed and transformed into the wild type strain CNO-1, which was cultured 28 ℃ for 2 days. Hyphal samples were examined by fluorescence microscopy, Scale bar = 10 µm. (D) Phenotypes of *FsTreA* mutants on minimal medium (MM) plates with trehalose as the sole carbon source for 3 days. (E) Trehalase activity quantification of secreted protein in *FsTreA* mutants. The conidial suspensions of all strains was harvested from PDA plates after 7 days and incubated in PDB medium for 3 days. The hyphae of all strains were harvested for extracting secreted protein. Values represent means ± SE of three biological replicates. Different letters indicate significant differences at *p* < 0.05 as measured by Duncan’s multiple comparisons test.

*3.6 FstreA positively regulates virulence by mobilizing trehalose within sugarcane tissues*

To assess the impact of FsTreA on the virulence of *F. sacchari*, we inoculated sugarcane plants with conidia from both the wild-type and *FsTreA* mutant strains. Plants inoculated with the Δ*FsTreA* mutant displayed milder PBD symptoms and a lower DSI than those inoculated with WT at 14 days post-inoculation (dpi) . In contrast, plants inoculated with the O-FsTreA mutant showed no significant difference from those inoculated with WT. The virulence of the complementation strain (C-Δ*FsTreA*) was restored by reintroducing a wild-type copy of *FsTreA*. Consistent with *in planta* inoculation assays, the Δ*FsTreA* mutant resulted in significantly smaller lesions on sugarcane leaves compared to the wild-type (Figure 7A-C). These results suggest that FsTreA positively regulates *F. sacchari* virulence.

Since FsTreA functions as an acid trehalase, we propose that its impact on *F. sacchari* virulence is related to the mobilization of extracellular trehalose. To verify this hypothesis, sugarcane tissues inoculated with *FsTreA* mutant strains were analyzed at 48 hours post-inoculation. The results indicated significantly higher trehalose accumulation in tissues inoculated with Δ*FsTreA*, followed by those inoculated with the wild type, with the least accumulation observed in tissues inoculated with O-FsTreA (Figure 7D). W**e** then **tested whether adding trehalose to infection sites can rescue the virulence defect of Δ*FsTreA*. The results showed that, compared to the control, exogenous application of trehalose at infection sites increased the DSI across all strains. In particular, the virulence of Δ*FsTreA* was significantly enhanced (Figure S8).** This demonstrates that FsTreA enhances *F. sacchari* virulence by mobilizing host-derived trehalose from sugarcane.


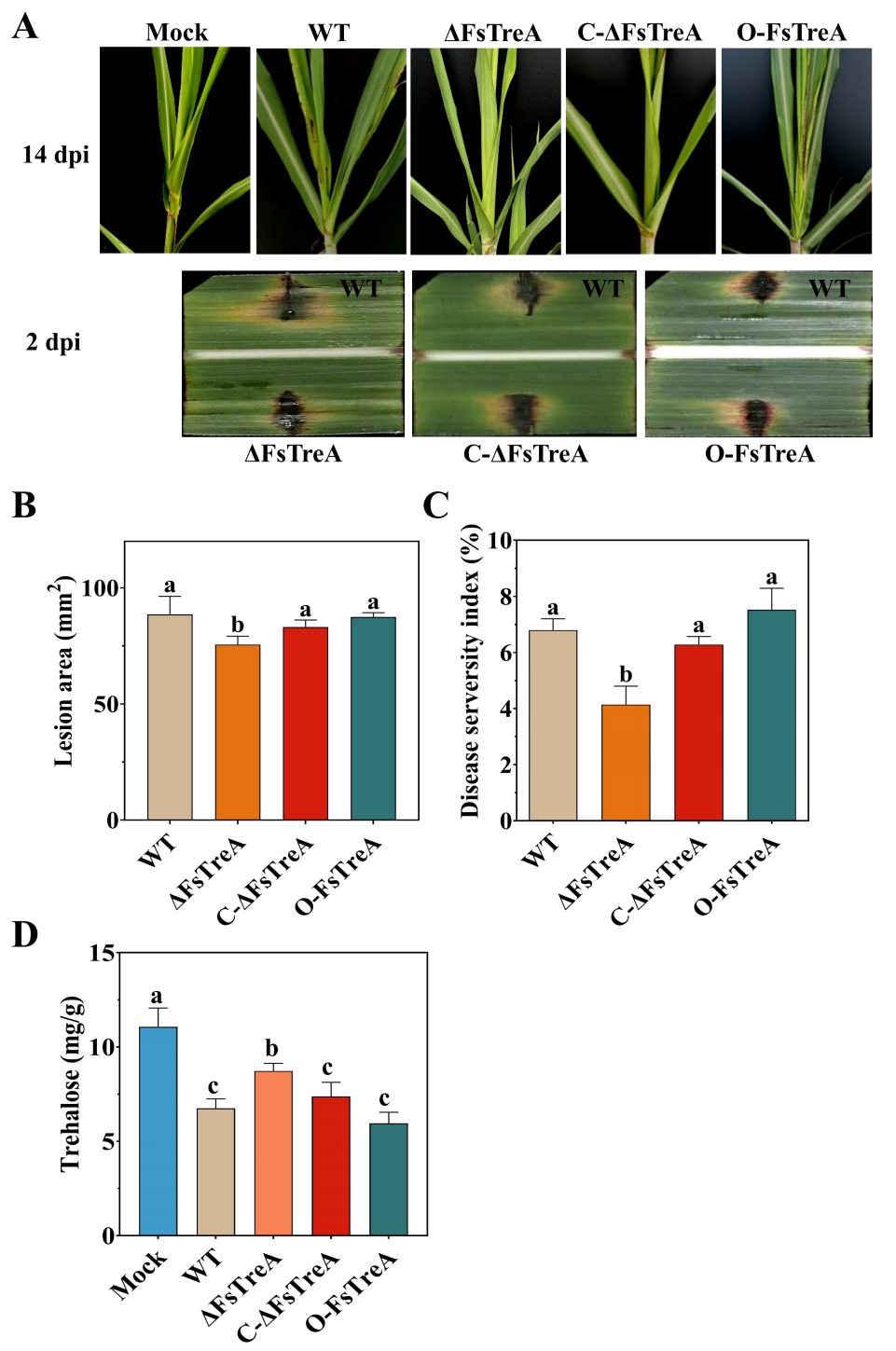


**Fig. 7** **FsTreA mobilizes trehalose from sugarcane tissue, contributing to the virulence of *F. sacchari*.** (A) Symptoms on sugarcane seedlings and leaves. Photographs were taken 14 dpi for the plant inoculation assays. For the leaf inoculation assays, representative photographs were taken at 48 hours post-inoculation (hpi). The notched leaf margin signifies the inoculation site of WT. (B, C) Quantification of disease severity index and lesion area. The disease severity index was determined using 50 seedlings per treatment. Lesion areas of 10 leaves per treatment were measured using ImageJ. (D) Trehalose quantification of sugarcane tissues inoculated with *FsTreA* mutant strains. Values represent means ± SE of three biological replicates. Different letters indicate significant differences at *p* < 0.05 as measured by Duncan’s multiple comparisons test.

In summary, FsTreA represents an evolutionarily adapted bifunctional trehalase in *F. sacchari*. Intracellularly, it coordinates trehalose mobilization in conjunction with neutral trehalase FsNth1 and acid trehalase FsAth1 through reciprocal compensation, thereby governing conidial germination. During host invasion, the secreted FsTreA utilizes extracellular trehalose from sugarcane as a pathogenic energy source, positively fueling fungal virulence (Figure 8).


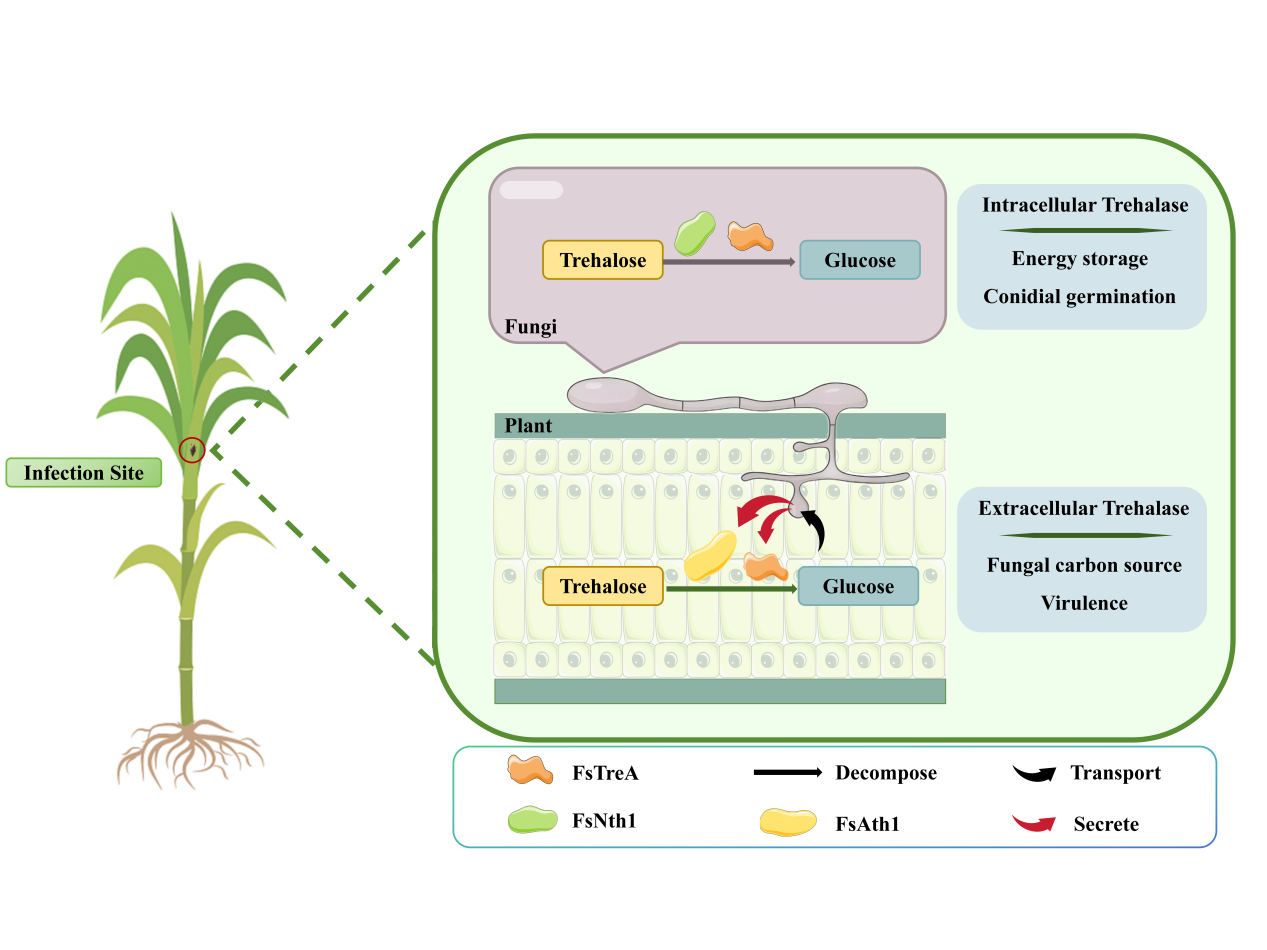


**Fig. 8** **Model of trehalase-mediated dual-compartment trehalose mobilization coordinating development and virulence in *F. sacchari*.**

1. **Discussion**

As the primary pathogen responsible for PBD, *F. sacchari* spreads via conidia to establish infection, with conidial germination being crucial for host successful colonization^[35]^. Trehalose is an important reserve carbohydrate that contributes to fungal energy requirements in cell processes such as sporulation, germination, and growth^[36]^. Although trehalases are known to regulate the mobilization of trehalose for conidial germination^[37-41]^, dormancy^[42, 43]^, abiotic stress resilience^[44-47]^, and virulence^[48-50]^, knowledge about trehalases' function in *F. sacchari* is scarce. Our study identified a unique trehalase FsTreA, distinct from the neutral trehalase FsNth1 and the acidic trehalase FsAth1, which exhibits bifunctional activity: coordinating intracellular trehalose metabolism and utilizing extracellular trehalose from sugarcane as an energy source to sustain virulence.

Trehalases facilitate fungal metabolism by hydrolyzing trehalose into two glucose molecules. Fungal trehalases are traditionally classified into neutral (cytosolic, GH37 family) and acidic (vacuolar/cell surface-localized, GH65 family) types^[51,52]^. Neutral trehalases like *S. cerevisiae* Nth1 possess a Ca²⁺- binding domain (CaBD) and conserved domain (CD), while acidic trehalases Ath1 only has a CD. Additionally, acid trehalases in pathogenic fungi possess an N-terminal signal peptide that directs their extracellular secretion, facilitating the utilization of exogenous trehalose as a carbon source, such as TRE1 in *Magnaporthe grisea* and *Gibberella zeae*^[21, 53]^. In this work, we classified the classic types of trehalases in *F. sacchari* as the acid and neutral trehalases as FsAth1 and FsNth1, respectively, assigning them into the GH65 and GH37 glycoside hydrolase families. Unexpectedly, a specific trehalase, FsTreA, represents a novel variant: it belongs to the GH37 family, but lacks the CaBD characteristic of neutral trehalases yet contains a signal peptide (SP) and a trehalase domain (Figure 1). Structurally, the coexistence of a GH37 catalytic domain and signal peptide in FsTreA provides a structural basis for its dual localization and function—enabling intracellular metabolic coordination and extracellular nutrient acquisition. This structure challenges the traditional dichotomous classification of fungal trehalases, indicating that *F. sacchari* has evolved a multifunctional trehalase to adapt to its pathogenic lifestyle. To elucidate its functional nature, we characterized the enzymatic properties of FsTreA. The pH profile revealed a broad pH range (pH 1.0-12.0) for FsTreA, peaking under acidic conditions (pH 3.0-4.0) while maintaining moderate activity under neutral conditions (pH 6.0-7.0), albeit lower than under acidic conditions. This pattern is consistent with its bifunctional nature (Figure S10A). Notably, its enzymatic activity was independent of Ca²⁺, distinguishing it from conventional neutral trehalases (Figure S10C). Furthermore, substrate specificity analysis confirmed that FsTreA exhibits high specificity for trehalose, supporting its role as a dedicated hydrolase (Figure S10B). Together, these findings illustrate how *F. sacchari* has evolved this multifunctional trehalase to adapt to its pathogenic lifestyle, integrating both structural innovation and biochemical versatility.

To further investigate the role of FsTreA in *F. sacchari*, we analyzed its expression pattern during conidial germination and hyphal morphogenesis. Unlike *FsAth1*, both *FsTreA* and *FsNth1* were significantly upregulated during this process, exhibiting peak expression at 6 hours, with the transcript level of *FsTreA remaining* higher than that of *FsNth1* (Figure 2). The results indicate that *FsTreA* and *FsNth1* contribute to the germination and growth of *F. sacch*ari. Then we engineered knockout (Δ*FsTreA*, ΔFsNth1 and ΔFsAth1), complementation (C-Δ*FsTreA*), and overexpression (O-FsTreA) mutant strains (Figure S4 & Figure S5). The significant depletion of trehalose in O-FsTreA spores indicates that FsTreA plays a role in mobilizing intracellular trehalose, similar to neutral trehalase in various fungi (Figure 5D)^[54, 55]^. In *Aspergillus niger*, deleting the intracellular trehalase gene *treB* resulted in fewer and less viable spores, and during the early stages of conidial germination, the internal levels of trehalose were higher than in the wild type^[56]^. These findings were similar to those of ΔFsNth1. Deletion of *FsNth1* resulted in severely impaired growth and development and reduced the germination rate to one-third of that in WT (Figure S11). However, the disruption of *FsTreA* markedly accelerated trehalose mobilization and enhanced germination and conidiation (Figure 4D). The results were contradictory to the expected phenotype of impaired trehalose mobilization. To explain this counterintuitive result, the expression of *FsNth1* and *FsAth1* in Δ*FsTreA* was quantified. The results showed that the expression of *FsNth1* and *FsAth1* in Δ*FsTreA* was significantly upregulated compared to WT. Conversely, the overexpression of *FsTreA* almost entirely suppressed *FsNth1* expression (Figure 5B). These results suggest that FsTreA and FsNth1 collaboratively regulate intracellular trehalose mobilization in *F. sacchari* via a reciprocal compensatory mechanism. This mechanism serves as a key adaptive trait for the fungus and provides an important insight into the regulation of fungal carbon metabolism.

Subsequently, we verified the ability of FsTreA to utilize extracellular trehalose. The yeast signal sequence trap and subcellular localization assay revealed that FsTreA can be secreted extracellularly and primarily localized to the cell surface. (Figure 6A-C). while enzymatic assays showed reduced extracellular trehalase activity in Δ*FsTreA* and elevated activity in O-FsTreA (Figure 6E). In conclusion, these results demonstrate for the first time that FsTreA functions as an acid trehalase capable of mobilizing extracellular trehalose, which is a critical trait for pathogens to acquire host nutrients. Intriguingly, emerging evidence suggests that trehalose from hosts acts as an important carbon source for many pathogens and can support their growth and colonization^[57]^. Despite the availability of exhaustive evidence documenting the biological benefits of trehalose in microbial pathogens, the precise mechanism by which host trehalose could be acquired by the filamentous fungi remains unknown. Critically, trehalase activity demonstrates a dose-dependent enhancement of virulence in FsTreA mutants, indicating that *F. sacchari* hijacks sugarcane trehalose to boost its infectious capacity, mirroring the carbon piracy strategy reported in *Phytophthora sojae*^[58]^.

Notably, O-FsTreA did not show enhanced virulence (Figure 7). We propose three hypotheses. First, secretory bottleneck: FsTreA secretion may reach saturation in the host; overexpression fails to enhance extracellular enzyme activity.For example, ER stress in rice downregulates PR proteins via *OsIRE1* to reduce secretory load and avoid overload^[59]^. Second, feedback inhibition: accumulated glucose from trehalose hydrolysis may trigger carbon metabolic feedback, such as glucose accumulation activating *CCR* in *M. oryzae*, thereby impairing the pathogen's capacity to develop infective structures like appressoria and invade host plants^[60]^. Third, host immune response: excess FsTreA may be recognized by sugarcane. Just as trehalase peptides from nematodes can activate the host MAPK response, FsTreA is likely to induce this MAPK-mediated response or other defense mechanisms, thereby offsetting its carbon acquisition advantage^[61]^. Further experiments—such as constructing FsTreA mutants with varying secretion efficiencies or detecting host defense gene expression—are needed to validate these hypotheses.

In summary, our results further reveal that FsTreA is a bifunctional trehalase in filamentous fungi: beyond its acid trehalase activity for extracellular trehalose mobilization, it also orchestrates intracellular trehalose metabolism in concert with FsNth1 and FsAth1. Together, these roles allow FsTreA to potentiate *F. sacchari* virulence. It provides a comprehensive insight into the function of trehalase and mechanisms in filamentous fungi, and expands understanding of pathogen-host nutrient competition. Furthermore, FsTreA makes it a potential target for PBD control due to its dual role in metabolism and its role in virulence, advancing the development of novel antifungal strategies. Our work enriches the understanding of pathogen-host nutrient competition and lays the foundation for further mechanistic studies on fungal pathogenicity.

**Funding**

This work was supported by the National Natural Science Foundation of China (32460642) and General Program of Guangxi Natural Science Foundation (2024JJA130132).

**Author statement**

The authors declare that the research was conducted in the absence of any commercial or financial relationships that could be construed as a potential conflict of interest.

**Acknowledgments**

We thank Profs. Baoshan Chen, Ziting Yao and Chengwu Zou for academic guidance, lab members for experimental assistance, and the State Key Laboratory for Conservation and Utilization of Subtropical Agro-Bioresources and Center for Sugarcane Bio Breeding for platform support.

**Data Availability**

The data that support the findings of this study are available from the corresponding author upon reasonable request.

**Reference**

1. Chen R, Zhao N. Sugarcane production and research in China[J]. International Sugar Journal, 2010, 112(1340):452, 454-457.
2. Vishwakarma S, Amrita N, Pradeep K, et al. Pokkah Boeng: an emerging disease of sugarcane[J]. Journal of Plant Pathology and Microbiology, 2013, 3(4):170.
3. Poorniammal R, Jernisha J, Prabhu S, et al. Sugarcane Pokkah Boeng Disease: Insights and future directions for effective management[J]. Life, 2024, 14(12):1533.
4. Lin Z, Xu S, Que Y, et al. Species-specific detection and identification of *Fusarium* species complex, the causal agent of sugarcane Pokkah Boeng in China[J]. PLOS ONE, 2014, 9(8): e104195.
5. de Carvalho M, Rocha K, de Melo M, et al. Identification and genetic diversity of *Fusarium sacchari* associated with Pokkah Boeng disease of sugarcane in Northeast Brazil[J]. Journal of Phytopathology, 2023, 171(1):36-47.
6. Vanaporn M, Sarkar-Tyson M, Kovacs-Simon A, et al. Trehalase plays a role in macrophage colonization and virulence of *Burkholderia pseudomallei* in insect and mammalian hosts[J]. Virulence, 2016, 8(1):30-40.
7. Bao Y, Akbar S, Yao W, et al. Genetic diversity and pathogenicity of *Fusarium fujikuroi* species complex (FFSC) causing sugarcane Pokkah Boeng Disease (PBD) in China[J]. Plant Disease, 2023, 107(5):1299-1309.
8. Ohtake S, Wang Y J. Trehalose: current use and future applications[J]. Journal of Pharmaceutical Sciences, 2011, 100(6):2020-2053.
9. D'Enfert C, Fontaine T. Molecular characterization of the *Aspergillus nidulans* treA gene encoding an acid trehalase required for growth on trehalose[J]. Mol Microbiol, 1997, 24(1):203-216.
10. Vanaporn M, Titball R W.Trehalose and bacterial virulence[J]. Virulence, 2020, 11(1):1192-1202.
11. Müller J, Boller T, Wiemken A.Trehalose and trehalase in plants: recent developments[J]. Plant Science, 1995, 112(1):1-9.
12. Sánchez-Fresneda R, Guirao-Abad J, Argüelles A, et al. Specific stress-induced storage of trehalose, glycerol and d-arabitol in response to oxidative and osmotic stress in *Candida albicans*[J]. Biochemical and Biophysical Research Communications, 2013, 430(4):1334-1339.
13. Paul M, Primavesi L, Jhurreea D, et al. Trehalose metabolism and signaling[J]. Annu Rev Plant Biol, 2008, 59(1):417-441.
14. Tapia H, Koshland D. Trehalose is a versatile and long-lived chaperone for desiccation tolerance[J]. Current Biology, 2014, 24(23):2758-2766.
15. Ocón A, Hampp R, Requena N. Trehalose turnover during abiotic stress in *arbuscular mycorrhizal* fungi[J]. New Phytologist, 2007, 174(4):879-891.
16. Doehlemann G, Berndt P, Hahn M. Trehalose metabolism is important for heat stress tolerance and spore germination of *Botrytis cinerea*[J]. Microbiology, 2006, 152(9):2625-2634.
17. Eleutherio E, Panek A, De Mesquita J, et al. Revisiting yeast trehalose metabolism[J]. Current Genetics, 2015, 61(3):263-274.
18. Shrestha P, Kim M, Elbasani E, et al. Prediction of trehalose-metabolic pathway and comparative analysis of KEGG, MetaCyc, and RAST databases based on complete genome of *Variovorax* sp. PAMC28711[J]. BMC Genomic Data, 2022, 23(1):4.
19. Ma W, Si H, Wan S, et al. The participation of trehalose metabolism in response to high-humidity stress in *Megoura crassicauda* (Hemiptera: Aphididae) [J]. Agronomy-Basel, 2024, 14(12):2958.
20. Chen A, Stadulis S, DeLeuze K, et al. Evaluating cellular roles and phenotypes associated with trehalose degradation genes in *Saccharomyces cerevisiae*[J]. G3: Genes Genomes Genetics, 2024, 14(11): jkae215.
21. Foster A, Jenkinson J,Talbot N.Trehalose synthesis and metabolism are required at different stages of plant infection by *Magnaporthe grisea*[J]. EMBO Journal, 2003, 22(2):225-235.
22. Pedreño Y, González-Párraga P, Martínez-Esparza M, et al. Disruption of the *Candida albicans* ATC1 gene encoding a cell-linked acid trehalase decreases hypha formation and infectivity without affecting resistance to oxidative stress[J]. Microbiology, 2007, 153(5):1372-1381.
23. Chen X, Zhang Z, Chen Z, et al. Potential antifungal targets based on glucose metabolism pathways of *Candida albicans*[J]. Front Microbiol, 2020, 11(11):296.
24. Meng J, Huang H, Li Y, et al. First report of *Fusarium sacchari* causing Sugarcane Pokkah Boeng in China[J]. Plant Disease, 2019, 104(5):1553.
25. Chen Y, Yao Z, Zhao L, et al. Redundant and distinct roles of two 14-3-3 proteins in *Fusarium sacchari*, pathogen of Sugarcane Pokkah Boeng Disease[J]. Journal of Fungi (Basel), 2024, 10(4):257.
26. Letunic I, Bork P. Interactive Tree of Life (iTOL) v6: recent updates to the phylogenetic tree display and annotation tool[J]. Nucleic Acids Research, 2024, 52(W1): W78-W82.
27. Watanabe M, Yonezawa T, Lee K, et al. Evaluation of genetic markers for identifying isolates of the species of the genus *Fusarium*[J]. Journal of the Science of Food and Agriculture, 2011, 91(13):2500-2504.
28. Rao X, Huang X, Zhou Z, et al. An improvement of the 2ˆ (–delta delta CT) method for quantitative real-time polymerase chain reaction data analysis[J]. Biostatistics, Bioinformatics and Biomathematics, 2013, 3(3):71.
29. Kistler H, Benny U. Genetic transformation of the fungal plant wilt pathogen, *Fusarium oxysporum*[J]. Current Genetics, 1988, 13(2):145-149.
30. Zhang W, Zhao W, Huang J, et al. PEG-mediated genetic transformation of *Fusarium oxysporum* f. sp. conglutinans to study pathogenesis in cabbage[J]. Chiang Mai Journal Science, 2014, 41:945-956.
31. Moradi M, Dehne H, Steiner U, et al. Improved procedure for mass inoculum production of *Fusarium* species in a short period of time[J]. Applied Entomology and Phytopathology, 2017, 84(2):21-31.
32. Yin W, Wang Y, Chen T, et al. Functional evaluation of the signal peptides of secreted proteins[J]. Bio-protocol, 2018, 8(9): e2839.
33. Su X, Lu G, Rehman L, et al. MCherry-labeled *Verticillium dahliae* could be utilized to investigate its pathogenicity process in *Nicotiana benthamiana*[J]. Genes (Basel), 2018, 9(10):508.
34. Liang H, Li F, Huang Y, et al. FsCGBP, a Cutinase G-Box Binding protein, regulates the growth, development, and virulence of *Fusarium sacchari*, the pathogen of Sugarcane Pokkah Boeng Disease[J]. Journal of Fungi, 2024, 10(4):246.
35. Jenkinson P, Parry D. Splash dispersal of conidia of *Fusarium culmorum* and *Fusarium avenaceum*[J]. Mycological Research, 1994, 98(5):506-510.
36. Lowe R, Lord M, Rybak K, et al. Trehalose biosynthesis is involved in sporulation of *Stagonospora nodorum*[J]. Fungal Genetics and Biology, 2009, 46(5):381-389.
37. Plabutong N, Ekronarongchai S, Niwetbowornchai N, et al. The inhibitory effect of validamycin a on *Aspergillus flavus*[J]. International Journal of Microbiology, 2020, 2020(1):3972415.
38. Van Mulders R,Van Laere A. Cyclic AMP, trehalase and germination of *Phycomyces blakesleeanus* spores[J]. Microbiology, 1984, 130(3):541-547.
39. Hagiwara D, Takahashi H, Kusuya Y, et al. Comparative transcriptome analysis revealing dormant conidia and germination associated genes in *Aspergillus* species: an essential role for AtfA in conidial dormancy[J]. BMC Genomics, 2016, 17(1):358.
40. Seong K, Zhao X, Xu J, et al. Conidial germination in the filamentous fungus *Fusarium graminearum*[J]. Fungal Genetics and Biology, 2008, 45(4):389-399.
41. Dong C, Ma M, Xue R, et al. Functional characterization of trehalases 1, 2, and 4 in *Nosema bombycis* (microsporidia)[J]. Microb Pathog, 2025, 208:107986.
42. Shleeva M, Trutneva K, Demina G, et al. Free trehalose accumulation in dormant *Mycobacterium smegmatis* cells and its breakdown in early resuscitation phase[J]. Front Microbiol, 2017, 8:524.
43. Miguel-Rojas C, Cavinder B, Townsend J, et al. Comparative transcriptomics of *Fusarium graminearum* and *Magnaporthe oryzae* spore germination leading up to infection[J]. mBio, 2023, 14(1): e2422-e2442.
44. Sánchez-Fresneda R, Martínez-Esparza M, Maicas S, et al. In *Candida parapsilosis* the ATC1 gene encodes for an acid trehalase involved in trehalose hydrolysis, stress resistance and virulence[J]. PLOS ONE, 2014, 9(6): e99113.
45. Djonovic S, Urbach J, Drenkard E, et al. Trehalose biosynthesis promotes *Pseudomonas aeruginosa* pathogenicity in plants[J]. PLoS Pathog, 2013, 9(3): e1003217.
46. Ribeiro G, de Holanda P, Eleutherio E. Trehalose promotes biological fitness of fungi[J]. Fungal Biology, 2024, 128(8):2381-2389.
47. Ahlgren K, Olsson C, Ermilova I, et al. New insights into the protein stabilizing effects of trehalose by comparing with sucrose[J]. Physical Chemistry Chemical Physics, 2023, 25(32):21215-21226.
48. Chen X, Abubakar Y, Yang C, et al. Trehalose phosphate synthase complex-mediated regulation of trehalose 6-phosphate homeostasis is critical for development and pathogenesis in *Magnaporthe oryzae*[J]. mSystems, 2021, 6(5): e46221.
49. Wilson R, Jenkinson J, Gibson R, et al. Tps1 regulates the pentose phosphate pathway, nitrogen metabolism and fungal virulence[J]. The EMBO Journal, 2007, 26(15):3673-3685.
50. Peng G, Jin K,Liu Y, et al. Enhancing the utilization of host trehalose by fungal trehalase improves the virulence of fungal insecticide[J]. Applied Microbiology and Biotechnology, 2015, 99(20):8611-8618.
51. Argüelles J. Physiological roles of trehalose in bacteria and yeasts:a comparative analysis[J]. Archives of Microbiology, 2000, 174(4):217-224.
52. Alblova M, Smidova A, Docekal V, et al. Molecular basis of the 14-3-3 protein-dependent activation of yeast neutral trehalase Nth1[J]. Proceedings of the National Academy of Sciences, 2017, 114(46): E9811-E9820.
53. Parrou J, Jules M,Beltran G, et al. Acid trehalase in yeasts and filamentous fungi:localization, regulation and physiological function[J]. FEMS Yeast Res, 2005, 5(6-7):503-511.
54. D'Enfert C, Bonini B, Zapella P, et al. Neutral trehalases catalyse intracellular trehalose breakdown in the filamentous fungi *Aspergillus nidulans* and *Neurospora crassa*[J]. Molecular Microbiology, 1999, 32(3):471-483.
55. Chen L, Ma X,Sun T, et al. VdPT1 encoding a neutral trehalase of *Verticillium dahliae* is required for growth and virulence of the pathogen[J]. International Journal of Molecular Sciences, 2024, 25(1):294.
56. Svanström Å,Melin P. Intracellular trehalase activity is required for development, germination and heat-stress resistance of *Aspergillus niger* conidia[J]. Research in Microbiology, 2013, 164(2):91-99.
57. Collins J, Robinson C, Danhof H, et al. Dietary trehalose enhances virulence of epidemic *Clostridium difficile*[J]. Nature, 2018, 553(7688):291-294.
58. Zhu X, Fang D,Li D, et al. *Phytophthora sojae* boosts host trehalose accumulation to acquire carbon and initiate infection[J]. Nature Microbiology, 2023, 8(8):1561-1573.
59. Hayashi S, Wakasa Y, Takaiwa F. Functional integration between defence and IRE1-mediated ER stress response in rice[J]. Scientific Reports, 2012, 2(1): 670.
60. Huang Z, Wang Q, Li Y, et al. A multilayered regulatory network mediated by protein phosphatase 4 controls carbon catabolite repression and de-repression in *Magnaporthe oryzae*[J]. Communications Biology, 2025, 8(1):130.
61. Iino E, Kadota Y, Maki N, et al. A trehalase-derived MAMP triggers LecRK-V–mediated immune responses in *Arabidopsis*[J]. Science Advances, 2025, 11(31): eadv8896.
