# Supplementary material for: Bifunctional trehalase FsTreA coordinates intracellular mobilization and extracellular utilization of trehalose to modulate virulence in Fusarium sacchari
Source: Appl Environ Microbiol. 2026 May 14;92(6):e00697-26. doi: 10.1128/aem.00697-26 (PMC13274424; doi:10.1128/aem.00697-26)
Supplement: Supplemental figures — Fig. S1 to S11. [file aem.00697-26-s0002.docx]

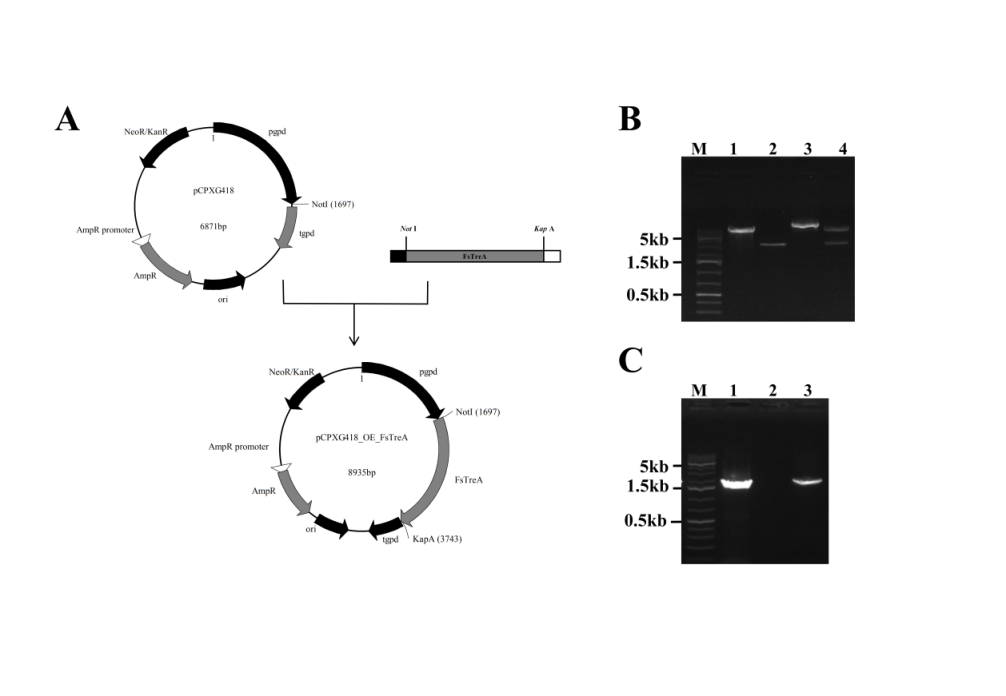


**Fig. S1 Generation of *FsTreA* overexpression mutant strain.** (A) Construction of the *FsTreA* overexpression plasmid. (B) Validation of the *FsTreA* overexpression plasmid, M: Marker; 1: pCPXG418 digested with *Not* Ⅰ and *Ksp* A, 2: *FsTreA-CDS* fragment, 3: pCPXG418-OE-FsTreA, 4: pCPXG418-OE-FsTreA digested with *Not* Ⅰ and *Ksp* A. (C) FsTreA overexpression mutants were validated by PCR using primers Pgpd-F / Tgpd-R. M: Marker; 1: pCPXG418-OE-FsTreA; 2: WT; 3: O-FsTreA.


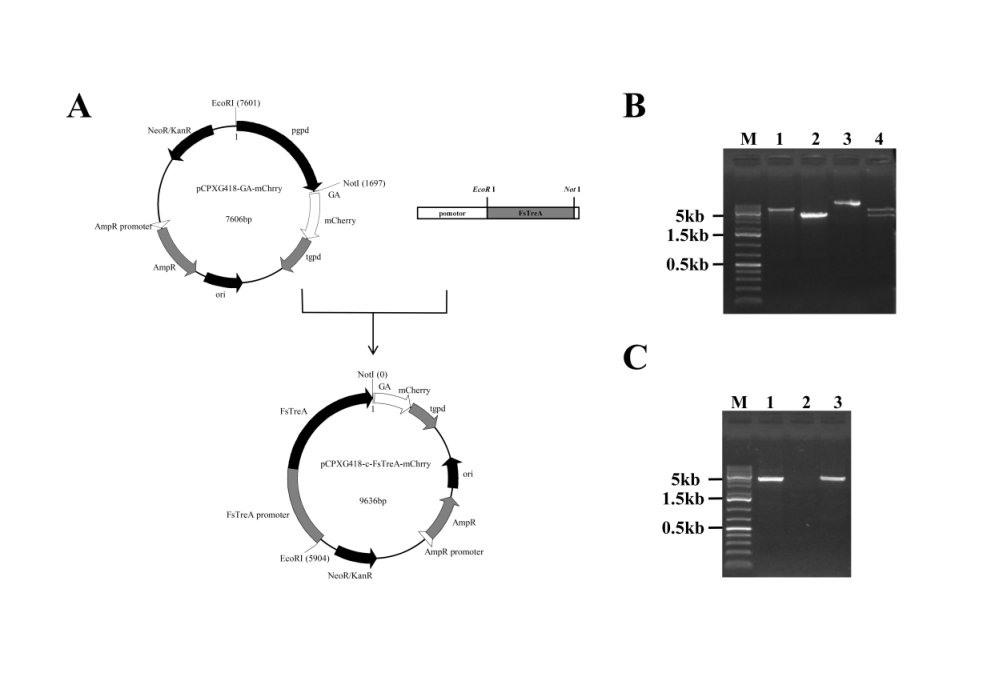


**Fig. S2 Generation of *FsTreA* complementation mutant strain.** (A) Construction of the *FsTreA* complementation plasmid. (B) Validation of the *FsTreA* complementation plasmid. M: Marker; 1: pCPXG418-mCherry digested with *EcoR* Ⅰ and *Not* Ⅰ ; 2: *FsTreA* complementation fragment; 3: pCPXG418-C-FsTreA-mCherry; 4: pCPXG418-C-FsTreA-mCherry digested with *EcoR* Ⅰ and *Not* Ⅰ. (C) Validation of FsTreA complementation mutants by PCR using primers FsTreA-Promoter-F / Tgpd-R. M: Marker; 1: pCPXG418-C-FsTreA-mCherry; 2: WT; 3: C-ΔFsTreA.


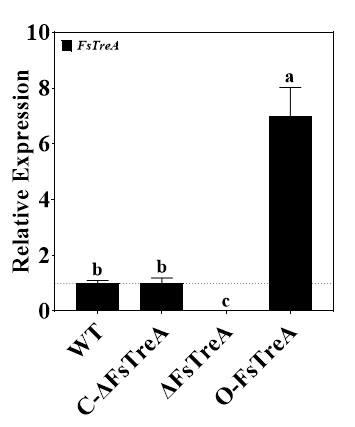


**Fig. S3 Validation of the FsTreA related mutant strains by RT-qPCR.** The expression of *FsTreA* transcripts was measured by quantitative real time RT-PCR (2^-ΔΔCT^ method) with 18S rRNA as internal reference. The transcript levels of WT was set to a value of 1.0. The data are presented as the means ± SE of three independent biological replicates. Different letters indicate significant different at *p* < 0.05 as measured by Duncan’s multiple comparisons test.


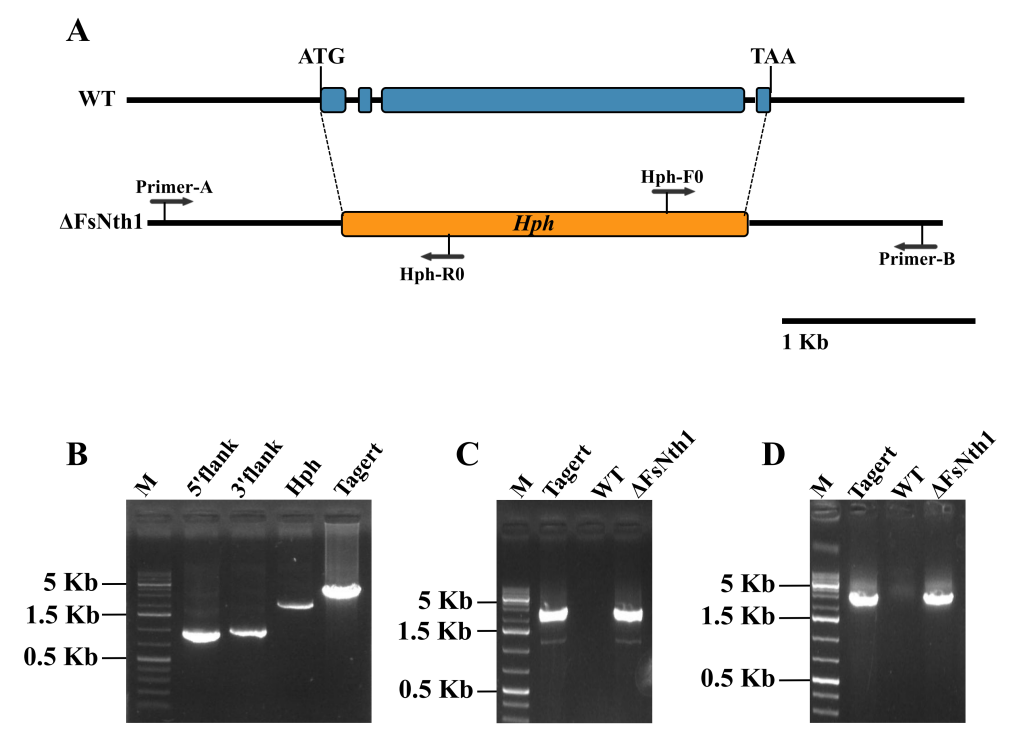


**Fig. S4 Generation of *FsNth1* deletion mutant strains.** (A, B) *FsNth1* gene locus and gene replacement construct. (C, D) *FsNth1* deletion mutants were validated by PCR with primer A / Hph-R0 and Hph-F0 / primer B, respectively.


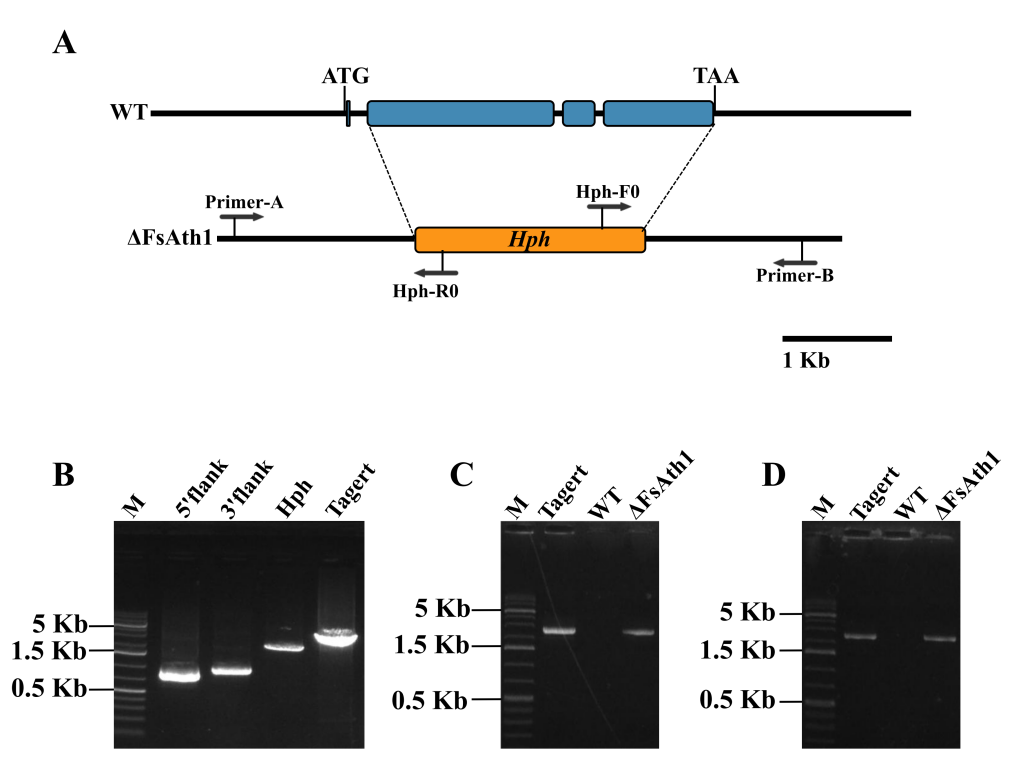


**Fig. S5 Generation of *FsAth1* deletion mutant strains.** (A, B) *FsAth1* gene locus and gene replacement construct. (C, D) *FsAth1* deletion mutants were validated by PCR with primer A / Hph-R0 and Hph-F0 / primer B, respectively.


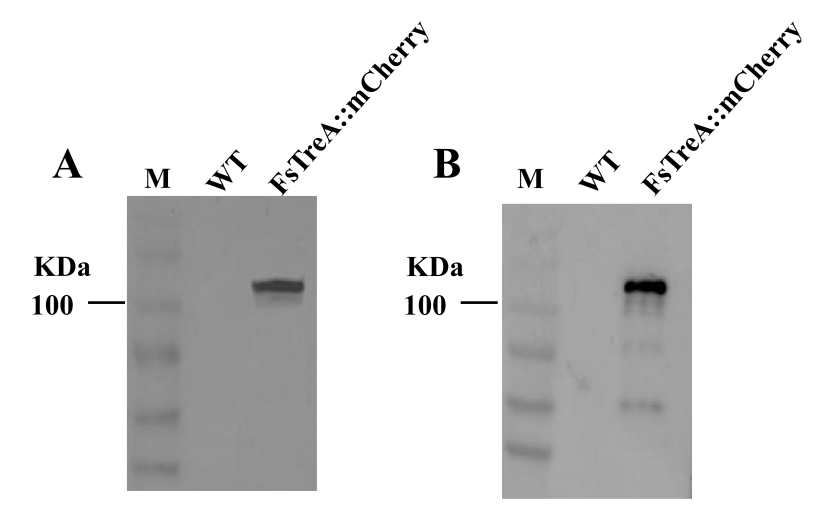


**Fig. S6 FsTreA is localized in both the cell wall and cytoplasm of *F. sacchari*.** (A) Western blotting verifies the localization of FsTreA in the cytoplasm of *F. sacchari* using Anti - mCherry. (B) Western blotting verifies the localization of FsTreA in the cell wall of *F. sacchari* using Anti - mCherry. The wild-type strain was set as a negative control.


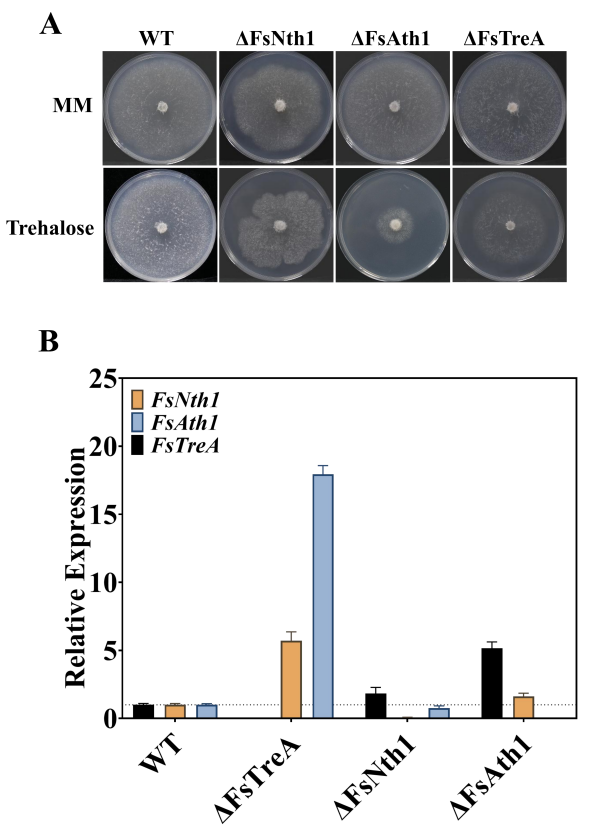


**Fig. S7 Phenotypic analysis of trehalase-encoding gene deletion mutants and expression patterns of trehalase-encoding genes in different deletion mutants of *F. sacchari*.** (A) Phenotypes of trehalases-encoding gene mutants on MM plates with trehalose as the sole carbon source for 7 days. (B) Expression patterns of three genes encoding trehalases in different deletion mutants. The expression of three genes was measured by RT-qPCR (2^-ΔΔct^ method) with 18S rRNA as internal reference. The three genes transcript level of WT was set to a value of 1.0.


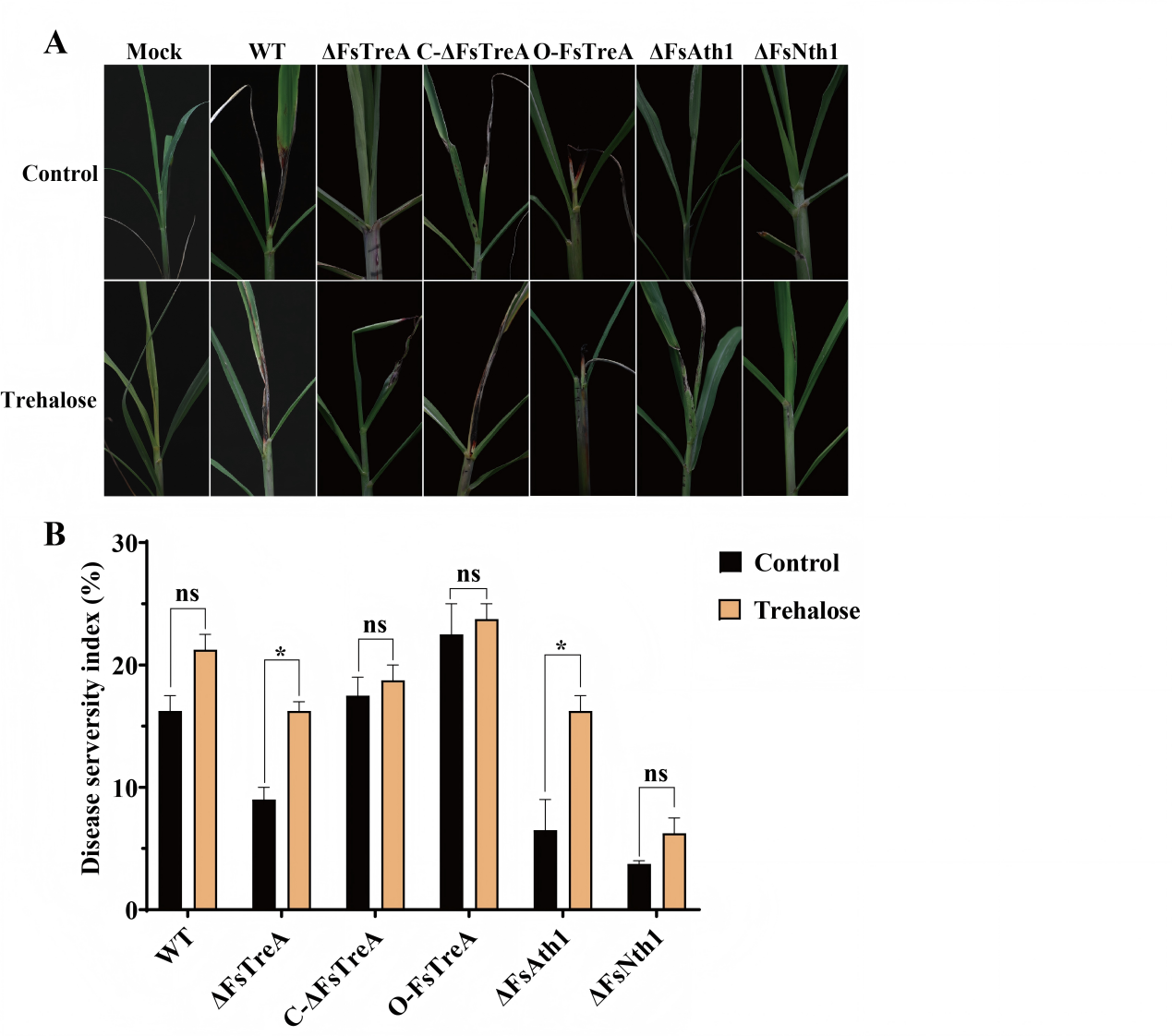


**Fig. S8 Trehalose serves as a nutritional source for *F. sacchari.*** (A) Symptoms of PBD on sugarcane seedlings. After inoculating each mutant strain, 20 mM trehalose was sprayed at the infection sites for 14 days, with water used as the control. Photographs were taken 14 days dpi for the plant inoculation assays. (B) Quantification of disease severity. The disease severity index was determined. Values represent means ± SE of fifty biological replicates. The statistical analyses were performed with Student's t test. **p* < 0.05, ns, no significant.


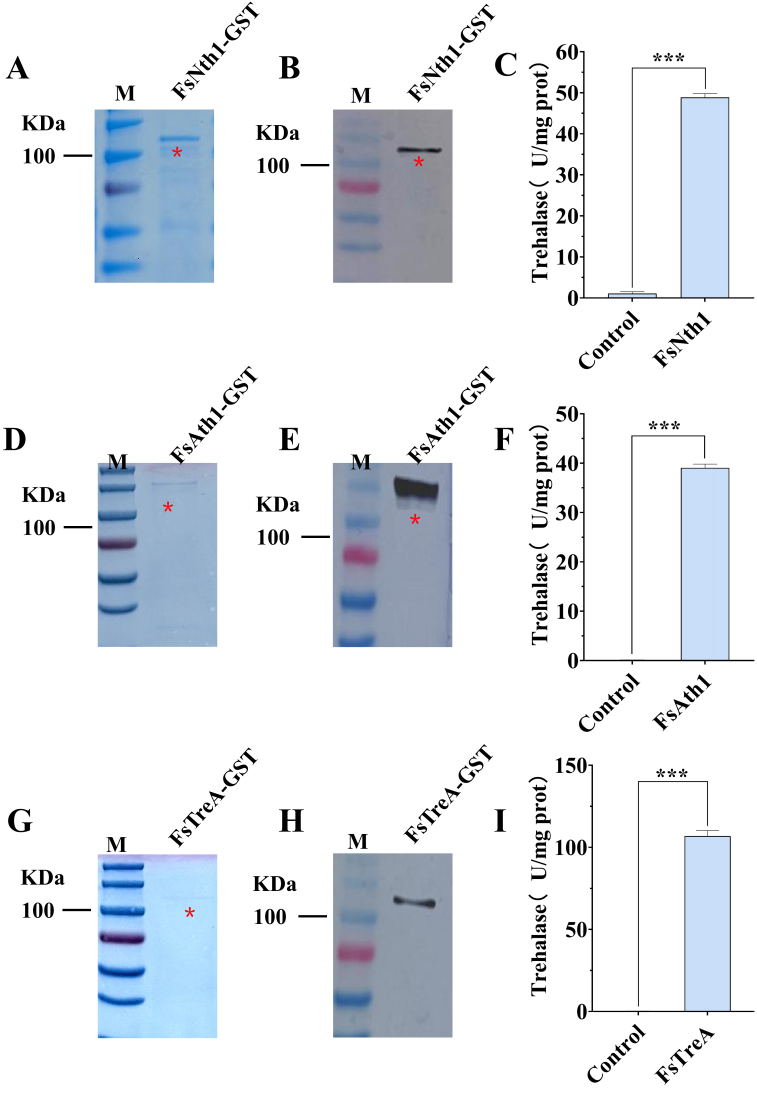


**Fig. S9 Expression, purification, and enzyme activity assay of trehalases.** (A, D, G) Identification of recombinant proteins FsNth1-GST, FsAth1-GST, and FsTreA-GST by SDS - PAGE, respectively. (B, E, H) Identification of recombinant proteins FsNth1-GST, FsAth1-GST, and FsTreA-GST by western blotting using Anti - GST, respectively. (C, F, I) The Trehalase activity quantification of recombinant proteins FsNth1-GST, FsAth1-GST, and FsTreA-GST, respectively. Denatured trehalases was used as the control. Values represent means ± SE of three biological replicates. The statistical analyses were performed with Student's t test. **p* < 0.05, ***p* < 0.01, ****p* < 0.001.


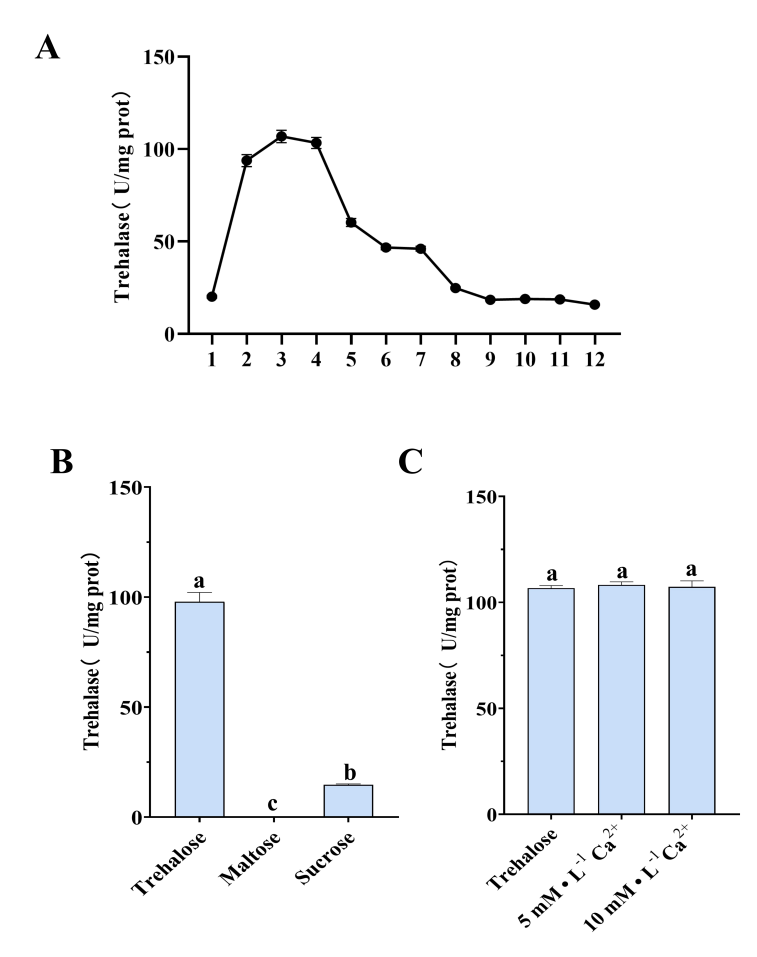


**Fig. S10 Enzymatic characterization of FsTreA.** (A) The trehalase activity of FsTreA under different pH conditions. (B) Substrate specificity verification of FsTreA. The activity of FsTreA was assayed at pH = 3, with the substrate buffer supplemented with 1% (w/v) trehalose, maltose, or sucrose, respectively. (C) Ca²⁺-dependency assays of FsTreA. The activity of FsTreA was assayed at pH = 3, with the substrate buffer supplemented with 0, 5 mM CaCl₂, and 10 mM CaCl₂, respectively. Values represent means ± SE of three biological replicates. Different letters indicate significant different at *p* < 0.05 as measured by Duncan’s multiple comparisons test.


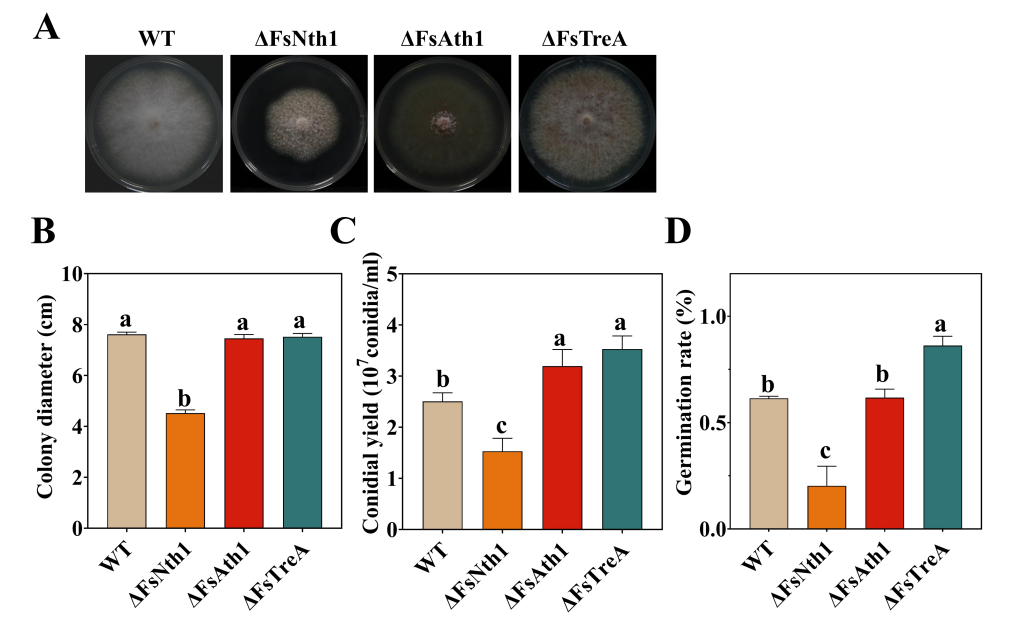


**Fig. S11 *FsNth1* regulates the growth and development of *F. sacchari.*** (A) Phenotypes of trehalases encoding gene deletion mutant strains. All strains were inoculated on PDA plates at 28℃ for 7 days. (B) Colony diameters of all strains were measured. (C) Statistics of conidial yield for all strains. Conidia were harvested from 7-days-old colonies on PDA plates. (D) Conidial germination rates in PDB at 28℃ for 6 hours. Values represent means ± SE of three biological replicates. Different letters indicate significant different at *p* < 0.05 as measured by Duncan’s multiple comparisons test.
