# Supplementary material for: Bifunctional trehalase FsTreA coordinates intracellular mobilization and extracellular utilization of trehalose to modulate virulence in Fusarium sacchari
Source: Appl Environ Microbiol. 2026 May 14;92(6):e00697-26. doi: 10.1128/aem.00697-26 (PMC13274424; doi:10.1128/aem.00697-26)
Supplement: Table S1 — Primers used in this study. [file aem.00697-26-s0003.docx]

**Table S1. Primers used in this study**

| **Primer** | **Primer sequence(5’-3’)** |
| --- | --- |
| FsTreA-A | ATTGACCGGCAGTTCTTGGT |
| FsTreA-B | TCTCCAGTCTCAAAGCGCAG |
| FsTreA-left-F | GTCTTAGGCGATGTATGTAT |
| FsTreA-left-R | TCCTTCTTTCTAGAGGATCCCCGGGTACCGCACAGCAGAATAGTTCCTC |
| FsTreA-right-F | TTCAATATCATCTTCTGTCGACCTGCAGGCGCTGAGAAGGCTATCCATAAG |
| FsTreA-right-R | GATGATGGCTAACTTGTGGTTATAC |
| Hph-Fo | GCAGCTATTTACCCGCAGGA |
| Hph-Ro | ACTGAGGAATCCGCTCTTGG |
| C-FsTreA- F | CAATATCATCTTCTGTCGACGAATTCTCCAGTGCCCAGCCAAAGAT |
| C-FsTreA- R | CAGCGCCTGCACCAGCTCCGCGGCCGCGAAATGAAACAGACGGCTAGACCTCT |
| FsTreA-qF | TTCCGGTCTACATCAAC |
| FsTreA-qR | CACCGCTATAAACCTCACCAGATGG |
| FsNth1-qF | AACTCGCACCTACTCCCAGG |
| FsNth1-qR | ACTTGAATCAAGAAGCGGCGGTT |
| FsAth1-qF | CACGACTATGAATGGCAGCG |
| FsAth1-qR | GTTCGCATAATAGTCGAGGTCATCC |
| OE-FsTreA*-F* | CTACTACTTTTAGAGGTACGCGGCCGCCCGTCTCCACGACATATCGC |
| OE-FsTreA*-R* | TCAGGTCAGCATGCGTTAACAAGCTTTTAGAAATGAAACAGACGGCTAGACCT |
| primer-A | ACTGATTTGTTATTCAATTGCTGTAGA |
| primer-B | CTCCATCTTTATAGTCCCATAAACACCTCAC |
| Pgpd-F | ACTAGTACCCGCCTTCATCGATG |
| Tgpd-R | AGGTGTTGGTCGCAGTCTAGGC |
| pSUC2-SP^FsTreA^-F | ATGCCGTCTCCACGACATATCGCTGCGGCTTTGGCTGCGTCAGCGACAACCGTTTCTGCT |
| pSUC2-SP^FsTreA^-R | AGCAGAAACGGTTGTCGCTGACGCAGCCAAAGCCGCAGCGATATGTCGTGGAGACGGCAT |
| 18s-F | TGGTTTCTAGGACCGCCGTA |
| 18s-R | CCTTGGCAAATGCTTTCGC |
